# A Circadian Clock-Regulated Toggle Switch Explains *AtGRP7* and *AtGRP8* Oscillations in *Arabidopsis thaliana*

**DOI:** 10.1371/journal.pcbi.1002986

**Published:** 2013-03-28

**Authors:** Christoph Schmal, Peter Reimann, Dorothee Staiger

**Affiliations:** 1 Condensed Matter Theory, Faculty of Physics, Bielefeld University, Bielefeld, Germany; 2 Molecular Cell Physiology, Faculty of Biology, Bielefeld University, Bielefeld, Germany; 3 CeBiTec, Bielefeld University, Bielefeld, Germany; University of Illinois at Urbana-Champaign, United States of America

## Abstract

The circadian clock controls many physiological processes in higher plants and causes a large fraction of the genome to be expressed with a 24h rhythm. The transcripts encoding the RNA-binding proteins *At*GRP7 (*Arabidopsis thaliana Glycine Rich Protein 7*) and *At*GRP8 oscillate with evening peaks. The circadian clock components CCA1 and LHY negatively affect *AtGRP7* expression at the level of transcription. *At*GRP7 and *At*GRP8, in turn, negatively auto-regulate and reciprocally cross-regulate post-transcriptionally: high protein levels promote the generation of an alternative splice form that is rapidly degraded. This clock-regulated feedback loop has been proposed to act as a molecular slave oscillator in clock output. While mathematical models describing the circadian core oscillator in *Arabidopsis thaliana* were introduced recently, we propose here the first model of a circadian slave oscillator. We define the slave oscillator in terms of ordinary differential equations and identify the model's parameters by an optimization procedure based on experimental results. The model successfully reproduces the pertinent experimental findings such as waveforms, phases, and half-lives of the time-dependent concentrations. Furthermore, we obtain insights into possible mechanisms underlying the observed experimental dynamics: the negative auto-regulation and reciprocal cross-regulation via alternative splicing could be responsible for the sharply peaking waveforms of the *AtGRP7* and *AtGRP8* mRNA. Moreover, our results suggest that the *AtGRP8* transcript oscillations are subordinated to those of *AtGRP7* due to a higher impact of *At*GRP7 protein on alternative splicing of its own and of the *AtGRP8* pre-mRNA compared to the impact of *At*GRP8 protein. Importantly, a bifurcation analysis provides theoretical evidence that the slave oscillator could be a toggle switch, arising from the reciprocal cross-regulation at the post-transcriptional level. In view of this, transcriptional repression of *AtGRP7* and *AtGRP8* by LHY and CCA1 induces oscillations of the toggle switch, leading to the observed high-amplitude oscillations of *AtGRP7* mRNA.

## Author Summary

The circadian clock organizes the day in the life of a plant by causing 24h rhythms in gene expression. For example, the core clockwork of the model plant *Arabidopsis thaliana* causes the transcripts encoding the RNA-binding proteins *At*GRP7 and *At*GRP8 to undergo high amplitude oscillations with a peak at the end of the day. *At*GRP7 and *At*GRP8, in turn, negatively auto-regulate and reciprocally cross-regulate their own expression by causing alternative splicing of their pre-mRNAs, followed by rapid degradation of the alternatively spliced transcripts. This has led to the suggestion that they represent molecular slave oscillators downstream of the core clock. Using a mathematical model we obtain insights into possible mechanisms underlying the experimentally observed dynamics, e.g. a higher impact of *At*GRP7 protein compared to the impact of *At*GRP8 protein on the alternative splicing explains the experimentally observed phases of their transcript. Previously, components that reciprocally repress their own transcription (double negative loops) have been shown to potentially act as a toggle switch between two states. We provide theoretical evidence that the slave oscillator could be a bistable toggle switch as well, operating at the post-transcriptional level.

## Introduction

Circadian clocks are endogenous timekeepers that can be found among all taxa of life [1]–[3]. They are able to generate stable oscillations with a period of approximately 24h that persist even under constant (free-running) conditions, i.e. in the absence of any rhythmic environmental influences that impact the clock. Entrainment by environmental signals such as light and temperature can synchronize the clock to the period of the Earth's rotation. Such a clockwork may confer a higher fitness to an organism as it allows to anticipate daily cycles of light and temperature in a spinning world [4], [5].

Circadian clocks are usually described as molecular networks including (interlocked) transcriptional - translational feedback loops [6]. In the higher plants model organism *Arabidopsis thaliana* an interplay of experiments and mathematical modeling shaped the current view on the circadian clock's network [7]–[13]. *Locke et al.* first modeled the structure of the circadian clock as a “*simple*” two-gene negative feedback loop [7], where the two partially redundant MYB transcription factors LATE ELONGATED HYPOCOTYL (LHY) and CIRCADIAN CLOCK ASSOCIATED 1 (CCA1) (combined to one variable LHY/CCA1) inhibit the transcription of their activator *TIMING OF CAB EXPRESSION 1* (*TOC1*). However, *in silico* and experimental mutant analysis revealed inconsistencies between the model and data [7], [8]. The assumed circadian clock architecture was therefore extended in successive steps [8]–[11] from this simple design to the idea of a clockwork that has a repressilator-like architecture at its core [13]. In this recent picture a “morning loop” consists of the morning-expressed genes LHY/CCA1 that activate the transcription of the *PSEUDO RESPONSE REGULATORS* 9, 7 and 5 (PRR9, PRR7 and PRR5) which in turn inhibit the transcription of *LHY/CCA1*. Furthermore, LHY/CCA1 is assumed to repress the transcription of the “evening loop” genes *EARLY FLOWERING 3* (*ELF3*) and *4* (*ELF4*), *LUX ARRHYTHMO* (*LUX*), *GIGANTEA* (GI), and *TOC1*, respectively. ELF3, ELF4 and LUX form a protein complex (evening complex, EC) that inhibits the transcription of *PRR9*, thereby connecting the evening loop with the morning loop, which closes the feedback loop circuitry [14].

The circadian clock affects many physiological processes in *Arabidopsis thaliana*, including the oscillation of free cytosolic calcium [15], stomatal opening, cotyledon and leaf movement [16], and even enables the plant to measure day-length, track seasons and thereby triggers the onset of flowering [17]. Underlying these physiological rhythms is a widespread control of gene expression by the circadian clock [18]. However, it is still not completely understood how the rhythmicity of the circadian clock is transmitted to its output genes. This may occur either directly by binding of clock proteins to their target genes or indirectly via signal transduction chains. One possibility to maintain the rhythmicity along such a signal transduction chain could be via slave oscillators that are driven by the circadian core oscillator and shape their oscillatory profile due to negative auto-regulation. *Colin Pittendrigh* already proposed in 1981 that “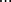 *any feedback loop in the organism is a potential slave oscillator, and if the circadian pacemaker can make input to the loop, the slave will assume a circadian period and become part of the temporal program that the pacemaker drives*” [19]. Genetic variation in such a slave oscillator can change its properties, e.g. the phase relation to the core oscillator, and thus the organisms' “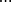 temporal program is open to evolutionary adjustment” [19] without the need for change in the core oscillator itself. Since driven by the core oscillator, the slave oscillator does not have to share all of the core oscillator's properties: It is not necessary that the slave oscillator exhibits independent self-sustaining oscillations, shows temperature compensation, or gains direct input from light [19], [20]. On the other hand, an indispensable pre-requisite of a slave oscillator is that it must not to act in any way back onto the core oscillator.

**Figure 1:**
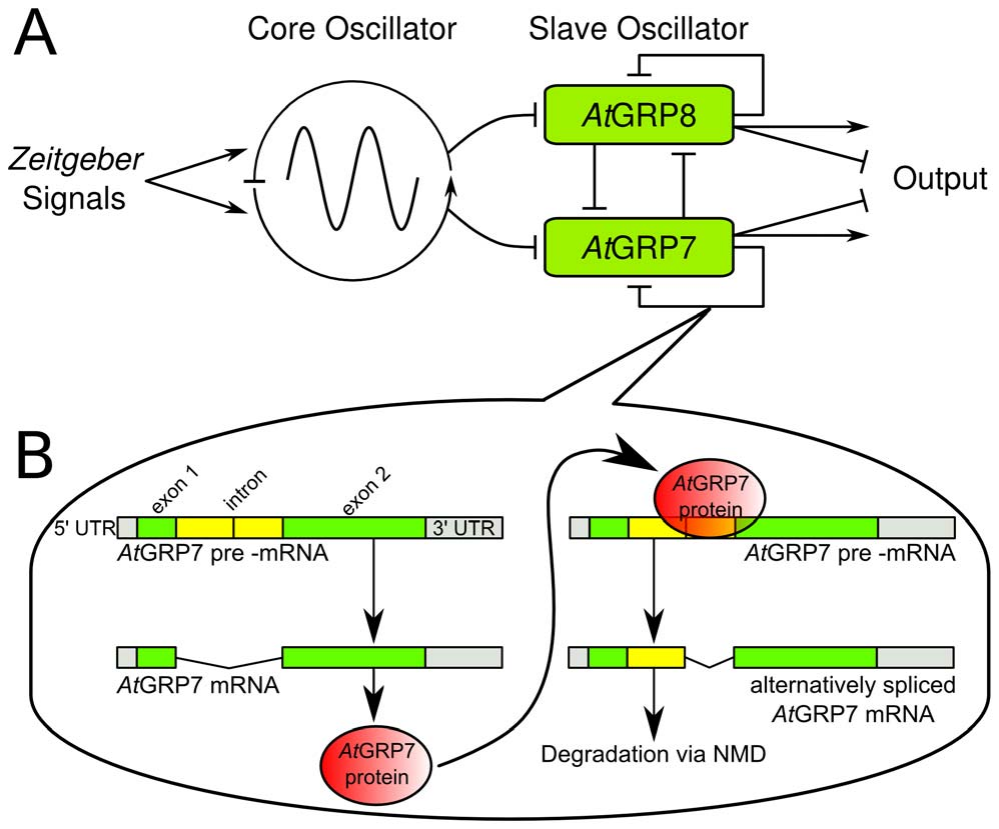
Proposed network structure and mechanism of *At*GRP7 and *At*GRP8 auto-regulation and cross-regulation. A) The circadian core oscillator is synchronized to the rhythm of a given external *zeitgeber* signal. It drives the slave oscillator composed of *At*GRP7 and *At*GRP8 (since the core oscillator genes LHY/CCA1 are assumed to inhibit the transcription of *AtGRP7* and *AtGRP8*). *At*GRP7 and *At*GRP8 negatively auto-regulate and cross-regulate each other. B) The negative auto-regulation and cross-regulation involves an alternative splicing mechanism coupled to NMD [73]: The *AtGRP7* pre-mRNA consists of two exons (green), separated by an intron (yellow) and bounded by the 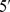 and 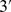 untranslated region (UTR) (gray). Its mature mRNA, with the intron completely spliced out, can produce functional protein (red). Both *At*GRP7 as well as *At*GRP8 protein can bind the *AtGRP7* pre-mRNA and induce the production of an alternatively spliced mRNA variant, retaining the first half of the intron. This alternatively spliced mRNA cannot produce functional protein due to a premature termination codon and is degraded via NMD.

The two RNA binding proteins *Arabidopsis thaliana Glycine Rich Protein* 7 and 8 (*At*GRP7 and *At*GRP8), also known as *Cold and Circadian-Regulated* 2 and 1 (*CCR2* and *CCR1*), respectively, have been proposed to represent such a molecular slave oscillator [21]–[23]. These proteins share 77 percent of sequence identity and contain an approximately 80 amino acid long RNA-recognition motif at the amino-terminus and a carboxy terminus mainly consisting of glycine repeats [21], [24]. The transcripts of both genes undergo circadian oscillations with evening peaks. The maximum of *AtGRP8* slightly precedes that of *AtGRP7* by 1–2 hours [25]. The *At*GRP7 protein oscillates with a four hour delay compared to its transcript [22]. In plants constitutively over-expressing CCA1 [26] or LHY [27], *AtGRP7* mRNA oscillations are dampened under constant light conditions, approaching the trough value of their corresponding oscillations in wild type plants, and thus suggesting that the transcription of *At*GRP7 is rhythmically repressed rather than activated by these partially redundant core oscillator genes. Apart from this transcriptional regulation *At*GRP7 also negatively auto-regulates the steady-state abundance of its own mRNA via a post-transcriptional mechanism [28]. When *At*GRP7 protein levels are high, an alternatively spliced transcript is produced at the expense of the fully spliced mRNA [22]. This alternative splice form is generated through the use of an alternative 5′ splice site and retains part of the intron. Due to a premature termination codon this alternatively spliced transcript cannot be translated into functional protein and is rapidly degraded via the nonsense-mediated decay (NMD) pathway [28], [29]. Since *At*GRP7 binds to its own transcript *in vitro* and *in vivo*, this alternative splicing likely is promoted by direct binding of *At*GRP7 to its own pre-mRNA [30], [31]. *At*GRP8 also auto-regulates itself and both proteins cross-regulate each other by the same mechanism. Our regulatory network is therefore composed of two auto-regulatory negative feedback loops, interlocked with each other and driven by the circadian core oscillator, as depicted in Figure 1.

Apart from the negative auto-regulation, *At*GRP7 affects the accumulation of a suite of circadian clock regulated genes in a time-of-day dependent manner, supporting the hypothesis that it acts as a slave oscillator between the core oscillator and the clock output: Rhythmic transcripts, whose steady state abundance is reduced upon *AtGRP7* overexpression, peak in the evening like *At*GRP7 itself, whereas rhythmic transcripts with an elevated steady state abundance peak 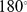 out of phase towards the morning [32].

Furthermore, it has been shown that *At*GRP7 has an impact on various other physiological processes: It promotes the floral transition [33], plays a role in the plants innate immune system [34], [35], and is known to mediate responses to stresses such as oxidative stress, high salt, mannitol, or cold [21], [36], [37].

Recently, various mathematical models for the circadian core oscillator in *Arabidopsis thaliana* have been developed [7]–[13]. In this paper we model the *At*GRP7 and *At*GRP8 feedback loops in terms of ordinary differential equations and thus propose the first mathematical model of a molecular slave oscillator in *Arabidopsis thaliana*. We note that a related model of a clock-controlled system has been put forward by *Salazar et al.* [38]. The molecular components of this system do not incorporate any feedback mechanism and are therefore unable to reshape their own oscillatory profile. Thus, they do not adopt all of the above mentioned specifications of a slave oscillator.

## Results/Discussion

### Modeling the *At*GRP7-*At*GRP8 Interlocked Feedback Loops

In order to model the essential layers of *At*GRP7 and *At*GRP8 regulation we need six dynamical variables, namely the concentrations of the pre-mRNA (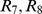), mRNA (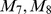), and protein (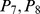) of *At*GRP7 and *At*GRP8. In the absence of any measured data that distinguish between cytoplasmic and nuclear protein concentrations, we, in particular, do not take into account that *At*GRP7 and *At*GRP8 localize to both the nucleus and the cytoplasm [39], [40], as it was done e.g., in [11]. The driving force of the *AtGRP7* oscillations is the periodic change in protein concentration of the core oscillator components LHY/CCA1, combined into one variable 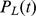. Throughout the first part of the paper we adopt the previously established mathematical model of *Pokhilko et al.* [11]. In principle, one could also use any other time periodic function or generic oscillator model that properly imitates the observed protein concentration 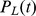 for a given experimental situation. Two examples of this type are a modified Poincaré oscillator and the refined model of *Pokhilko et al.* [13] as considered towards the end of our paper (see section *Robustness Against Variations in the LHY/CCA1 Protein Oscillations*).

**Figure 2:**
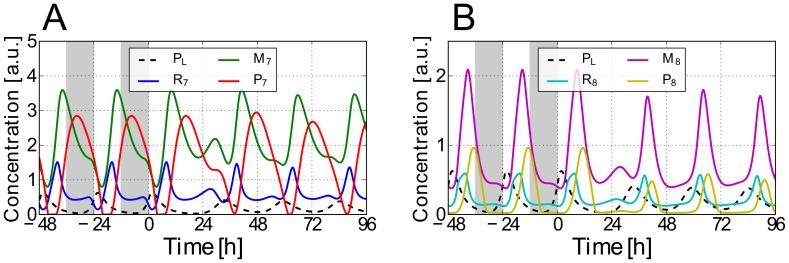
Systems dynamics for the “optimal” parameter set under 12h∶12h LD and LL conditions. *Solid lines* denote solutions of equations (1)–(6) for the “optimal” parameter set from Table 1: A) *At*GRP7 pre-mRNA (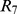), mRNA (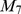), and protein (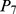) concentrations. B) *At*GRP8 pre-mRNA (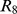), mRNA (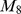), and protein (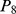) concentrations. *Dashed lines* denote the protein concentration 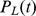 of the core oscillator gene LHY/CCA1. Shown are the last two days in 12h∶12h LD conditions (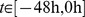) and the first four days after switching to constant light conditions (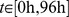). Throughout this paper, a gray-shaded background indicates darkness.

The original model provided by *Pokhilko et al.* [11] involves 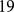 dynamical variables and 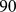 parameters whose quantitative values are taken over from that paper. Likewise, we utilize the same specific initial conditions for the core oscillator as in [11]. The externally imposed light input consists of either constant light (LL) or diurnal conditions such as 12 hours of light and 12 hours of darkness (abbreviated as 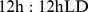) or 8 hours of light and 16 hours of darkness (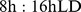), also denoted as short day conditions. These light conditions enter our core oscillator dynamics as detailed in [11] (especially continuous transitions instead of binary, i.e. on–off, light-dark transitions are used). Typical examples of the protein concentrations 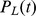 obtained in this way are depicted as dashed lines in Figure 2. In view of the fact that the *AtGRP7* mRNA steady state abundance seems not to be light-induced (unpublished data) we assume no direct light effect on the slave oscillator. This assumption is also coherent with Pittendrigh's definition, proposing that the slave oscillator could receive the light input only indirectly via the core oscillator [19].

Given the input 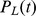 of the core oscillator to the *At*GRP7 and *At*GRP8 feedback loops, we model the temporal evolution of the slave oscillator's dynamical variables 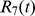, 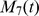, 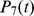, 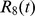, 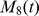, and 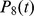 as follows
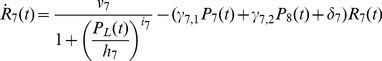
(1)

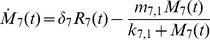
(2)

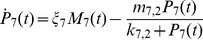
(3)

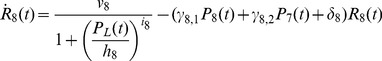
(4)

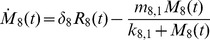
(5)

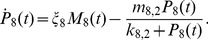
(6)


Consistent with other circadian clock models [7]–[11], in the first term on the right-hand-side of equation (1) we use a sigmoidal Hill repressor function, describing the negative regulation of *AtGRP7* transcription by LHY/CCA1. The pertinent transcription rate 
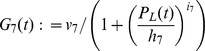
 of *AtGRP7* is then given in terms of the maximal transcription rate 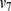, the Hill coefficient 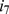, the activation coefficient 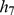, and the LHY/CCA1 protein concentration 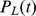. The loss term in equation (1) describes the normal and alternative splicing of *AtGRP7* pre-mRNA. It is assumed that the *AtGRP7* pre-mRNA is either spliced into its mature mRNA or into its alternative splice form, without considering any further degradation pathway. The kinetics for the splicing of the *AtGRP7* pre-mRNA into its alternative splice form, promoted by the binding of *At*GRP7 protein to its own pre-mRNA, is assumed to depend on the splicing coefficient 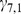 and the concentrations of the *At*GRP7 pre-mRNA (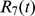) and protein (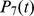). Equivalent kinetics are used for the alternative splicing of *AtGRP7* pre-mRNA promoted by the binding of *At*GRP8 protein. Note that 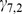 is the coupling parameter between *At*GRP8 and *At*GRP7, i.e. the impact of *At*GRP8 on alternative splicing of the *AtGRP7* pre-mRNA. The normal splicing of *AtGRP7* pre-mRNA into its mature mRNA is supposed to depend on a splicing coefficient 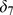 as well as the pre-mRNA concentration 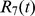 and appears as the gain term in the first part of equation (2). The second part of equation (2) describes the mRNA degradation as Michaelis-Menten kinetics that account for saturation by means of the Michaelis constant 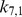 and the maximal degradation rate 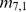. A similar Michalis-Menten degradation appears in equation (3), while 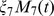 describes the translation of mRNA into protein. Analogous considerations apply to equations (4)–(6), modeling *At*GRP8. As usual, all the kinetic parameters in (1)–(6) are tacitly restricted to positive real values.

Collecting all 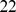 kinetic parameters into a vector 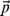 and the six dynamical variables into a vector 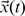 with components 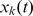, 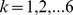, equations (1)–(6) can be written in the form of a parameterized non-autonomous dynamical system
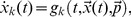
(7)where the explicit dependence on time 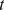 is a consequence of the external driving term 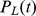 in (1) and (4).

### Parameter Estimation

In analogy to [7], we use the value 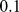 as initial conditions for all six dynamical variables in (1)–(6). Then, we numerically solve equations (1)–(6) for 14 days under 12 h∶12 h LD (entrainment) conditions followed by 13 days under constant light (free-running) conditions (see *Methods* for further details). In general the solutions are different for every parameter set 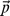. As it is often the case in biological modeling, none of these parameters is known from experiments [7]–[10]. So, the remaining challenge is now to identify the specific parameter set for which the solution reproduces as well as possible the following known (sparse and often noisy) experimental findings: 1. Both transcripts perform periodic oscillations with the same period as the core oscillator, both under LD and LL conditions [25]. 2. The transcript oscillations exhibit evening peaks with the peak of *AtGRP8* preceding that of *AtGRP7* by approximately 1–2 hours [22], [25], [29]. The corresponding *At*GRP7 protein concentrations oscillate with an approximately four hour delay compared to the transcript [22]. 3. The amplitudes of their oscillations are roughly comparable to those of the core oscillator [25]. 4. The waveform of the mRNA and protein oscillations have been characterized by means of experimental time series [22], [23], [25]. 5. *AtGRP7* mRNA is reduced to 50% within 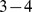 hours after experimentally suppressing its transcription [28].

In order to find an optimal parameter set, we defined a cost function 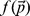 (described in detail in Text S1 A) which quantifies the deviation of the corresponding solution from these experimental findings 1–5 for every given parameter set 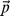. In a next step we minimized this cost function 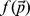 with respect to 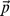.

**Figure 3:**
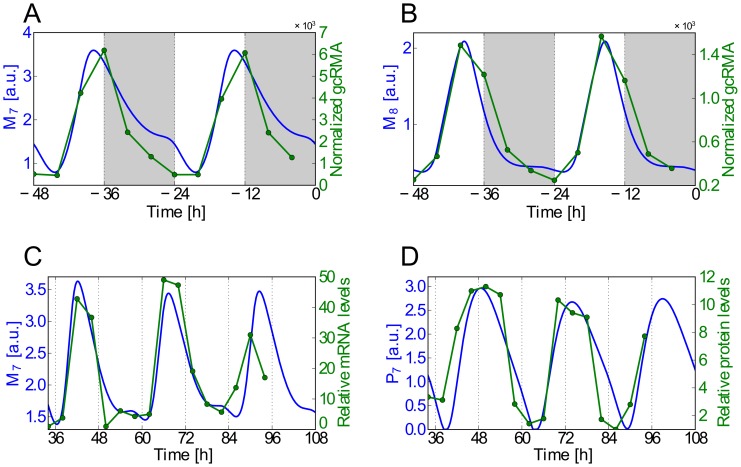
The model properly fits experimental data. A) Simulated *AtGRP7* and B) *AtGRP8* mRNA oscillations under 12h∶12h LD conditions (blue curves) are plotted together with the corresponding “COL_LDHH” experimental data set from the DIURNAL database (green curves with markers indicating data points), which uses *Columbia* wild type plants investigated under 12h∶12h LD entrainment condition with a constant temperature of 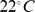. The DIURNAL database collects circadian microarray time series data based on Affymetrix chips and was normalized using gcRMA [25]. C) Simulated *AtGRP7* mRNA and D) protein oscillations under LL conditions, after entrainment under 8h∶16h LD conditions, are plotted together with the corresponding RNA and protein gel blot data taken from [22]. In [22], this gel blot data was published relative to the minimal level, which was defined as 1. Note that the time axis of the experimental data was adjusted by +34 hours. This takes into account a shortcoming of the core oscillator model adopted from [11], namely that the phase of the simulated *LHY/CCA1* mRNA oscillations under LL conditions in this core oscillator model only agrees with the corresponding data in the DIURNAL database (data sets “LL12_LDHH” and “LL23_LDHH” in [25]), if the time axis of those experimental data is adjusted by approximately ten hours. Since the samples in the experiments [74], [75] underlying these data sets were collected on days two and three after transferring the plants to LL conditions, we also did not take into account the first day in LL, altogether thus amounting to a total time-adjustment of +34h. Overall, the agreement between the simulated and experimental phases, periods, and waveforms is very good.

The detailed optimization procedure is described in *Methods*. Here we only summarize the main steps: To take into account the similarity of the two paralogous proteins *At*GRP7 and *At*GRP8 we first sampled the parameters for a reduced system, only consisting of *At*GRP7, using two million *Antonov-Saleev* quasi-random parameter sets. The network motif was then extended to the complete interlocked feedback loop structure, including also *At*GRP8. The parameters were chosen in order to generate two identical oscillatory profiles for *AtGRP7* and *AtGRP8*. The best one hundred solutions were then further optimized in the local neighborhood of a given parameter set using a *Nelder Mead* downhill simplex algorithm [41]. This modified sampling and optimization method led to better results than the full parameter space sampling and optimization, i.e. the best solutions have a lower cost function value and thus better fit the experimental data (compare Figure 3 A (discussed in the next section) and Figure S1). It might also reflect a possible evolutionary origin of that network motif since the high sequence similarity of *At*GRP7 and *At*GRP8 suggests that these genes are paralogues, originating from a gene duplication event [42], [43].

### Comparison with Experimental Results and Computational Predictions

#### *In silico* waveforms and phases are consistent with the experimental data

The simulations for the best parameter set found by our optimization scheme are shown in Figure 2 and the corresponding optimal parameter set is provided in Table 1.

**Table 1:** Optimal parameter set.

Description	Parameter	Value	Parameter	Value
Hill Coefficient	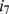	2.78	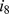	0.8
Maximal Transcription Rate	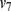	2.38	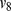	2.13
Activation Coefficient	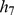	0.35	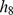	0.36
Alternative-Splicing Coefficient (Auto-Regulation)	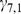	1.61	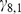	0.63
Alternative-Splicing Coefficient (Cross-Regulation)	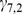	0.53	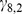	3.86
Normal-Splicing Coefficient	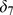	0.91	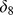	1.85
Maximal mRNA Degradation	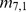	1.39	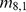	2.10
Michaelis Constant	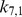	2.99	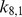	2.93
Translation Rate	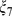	0.38	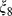	0.32
Maximal Protein Degradation	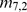	0.82	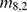	0.49
Michaelis Constant	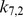	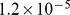	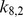	0.06

“Best” parameter set found by our optimization scheme: The Hill coefficients 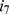 and 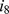 are unit-less positive real numbers. 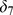, 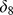, 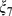, and 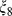 are rate constants for splicing and translation in units 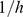. The activation and Michaelis constants in units of concentrations are 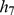, 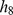, 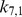, 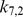, 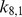, and 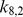. The maximal transcription and degradation rates 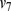, 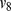, 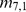, 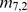, 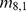, and 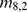 have units of concentration per hour. The alternative splicing coefficients 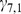, 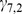, 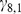, and 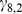 are given in units of the inverse of concentration times hour. As we cannot deduce explicit single cell concentration values from the experimental time traces used here, concentration values are given in arbitrary units ([a.u.]) rather than in some hypothetically defined absolute units.

As a first prominent quantity we consider the phase 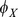 of an oscillating concentration 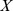 in units of *zeitgeber time* (zt), i.e. 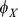 is defined as the time an oscillation needs to reach its maximal concentration after the onset of light in the external light-dark cycle. The *AtGRP7* mRNA peak under 12h∶12h LD conditions at 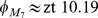 is very close to the phase estimated from the literature [25] and predetermined by the cost function (see Text S1 A). The *AtGRP8* mRNA peak at 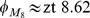 precedes that of *AtGRP7* by approximately 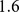 hours as previously shown experimentally [25]. The *At*GRP7 protein concentration is maximal at about 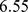 and 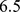 hours after the mRNA's peak in LD and LL, respectively, which is close to the literature value [22] (note that in [22] the relative protein concentrations were measured under LL conditions after entrainment in 8h∶16h LD conditions but the time span between the mRNA and the protein concentration peaks may be also a good approximation under the 12h∶12h LD conditions used in our simulations). Since there is no published experimental data on the *AtGRP7* and *AtGRP8* pre-mRNA as well as *At*GRP8 protein time traces, their corresponding simulations in Figure 2 can be considered as a first theoretical prediction of our present work.

A direct comparison between our simulated and the experimental time traces [22], [25], as depicted in Figure 3, shows that the proposed model mimics the experimentally observed phases, periods, and waveforms very well. The “shoulder” observed during the declining phase of the simulated *AtGRP7* mRNA concentration in Figure 3 A can sometimes be seen in experiments as well; e.g. in the data set of the DIURNAL database measured under 16h∶8h LD conditions (“long day” data set in [25]). In our simulations, the shape of this shoulder depends on the broadness and amplitude of the driving LHY/CCA1 protein oscillations. 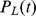 oscillations with a lower peak concentration, e.g. for simulations under LL conditions, lead to higher 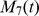 trough values and a less pronounced “shoulder“ (as one can see in Figures 2 and 3 C) due to the reduced transcriptional repression by LHY/CCA1. In experimental papers, not much attention has been paid so far to this fact but it actually could hint to the two-step transcriptional and post-transcriptional regulation of *At*GRP7 (see below). A further interesting feature of the system is the fact that the peak concentration of *AtGRP8* mRNA is always lower than the one of *AtGRP7* mRNA (see Figure 2) which was not taken into account by our cost function (see section *Parameter Estimation*) but is actually observed in experiments [25]. Furthermore, the trough values of 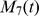 and 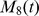 are always non-zero. This is consistent with experimental data given in [25], where a non-zero trough value was detectable among all data sets.

#### Importance of negative auto-regulation and reciprocal cross-regulation

Our simulations also support the assumption that the *At*GRP7 and *At*GRP8 negative auto-regulation and reciprocal cross-regulation could be responsible for the experimentally observed phases and sharply peaking waveforms of the *AtGRP7* and *AtGRP8* mRNA oscillations. The *AtGRP7* and *AtGRP8* pre-mRNA and mRNA concentrations reach their trough value soon after the rise of the LHY/CCA1 protein peak and quickly recover while LHY/CCA1 is declining. Subsequently, their concentrations start to fall again although the LHY/CCA1 protein concentration is still at its trough (see Figure 2). For the network topology proposed in Figure 1 A, this is only possible due to the negative auto- and cross-regulation in equations (1)–(6): Elevated protein levels promote the generation of the alternative splice forms at the expense of mature mRNA. Upon reducing the impact of the negative auto-regulation of *AtGRP7* by gradually decreasing the (alternative) splicing coefficient 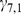, we observe a phase shift of the *AtGRP7* pre-mRNA and mRNA oscillations to a later time of day, see Figure S2. On top of that, the peaks of the 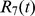 and 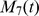 oscillations get increasingly broader and the previously observed ”shoulder“ of the *AtGRP7* mRNA as well as the second trough in the *AtGRP7* pre-mRNA progressively disappear. It is thus intuitively quite plausible that both shoulder and trough have their roots in the two-step transcriptional repression by LHY/CCA1 and the post-transcriptional auto-regulation of *At*GRP7. The importance of cross-regulation for the observed *AtGRP8* mRNA phase will be further discussed in the paragraph *AtGRP8 oscillations appear to be subordinated to AtGRP7*.

#### *In silico* half-life experiments

As a next quantity we consider the half-life of *At*GRP7 and *At*GRP8 mRNA and protein. As detailed in Text S1 A, we defined the simulated half-life 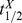 of a given species 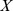 exactly along the lines of a previous experiment: The decay of a given species 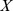 is measured after its production is interrupted, e.g. in the case of *AtGRP7* mRNA by transferring the plants to a medium supplemented with *cordycepin* which inhibits the RNA synthesis [28], [44]. A graphical illustration of the simulated half-life measurements can be seen in Figures S3 A/B.

Since in equation (2) the production of *AtGRP7* mRNA depends on the normal splicing 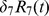 of its pre-mRNA to the mRNA and since its degradation kinetics are of Michaelis-Menten type, its half-life will depend on the initial conditions of the system. It will therefore vary, depending on the day time at which the transcription is interrupted (see Figures S3 C/D). This prediction could be tested in experiments, where transcriptional blockers, such as *cordycepin* and *actinomycin D*, are supplied at different phases of the day followed by a subsequent half-life determination.

The half-life 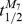 of 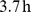, obtained after the interruption of RNA synthesis two hours before the 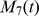 maximum is expected, is in good agreement with the corresponding experiment in [28] that has found a half-life between three and four hours. An analogous analysis for the *AtGRP8* half-life predicts a half-life 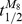 of 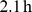. This shorter half-life of *AtGRP8* compared to that of *AtGRP7* can be partially explained by the smaller amplitude and lower peak concentrations of the 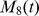 oscillations. The *AtGRP8* half-life has not been measured experimentally.

In order to measure the protein half-life *in silico* we set the parameters 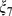 and 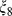 in equations (3) and (6) to zero, corresponding to an inhibition of protein translation. The resulting decoupled equations 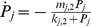 for 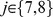 can be solved analytically. The half-life for a given initial value 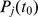 reads as
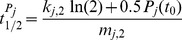
(8)and therefore depends on the initial value 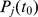, in contrast to the half-lives resulting from linear degradation kinetics. The protein half-lives over a full cycle under 12h∶12h LD conditions are shown in Figure S3 E/F. They change over the course of day and their highest values 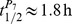 and 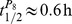 coincide with the protein concentration maxima at 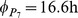 and 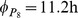, respectively.

#### *At*GRP8 oscillations appear to be subordinated to *At*GRP7

As described above (see end of section *Parameter Estimation*) we used an optimization scheme that mimics the possible evolutionary origin of the *At*GRP7 and *At*GRP8 interlocked feedback loops, namely a gene duplication followed by further evolution. Moreover, we assumed that *At*GRP7 and *At*GRP8 behave similarly (see Text S1 A). The cost function only takes into account two differences between them, namely an earlier peak of *AtGRP8* mRNA compared to *AtGRP7* mRNA and the fact that the *AtGRP8* mRNA half-life is not known. The optimization then leads to a model that proposes splicing coefficients that fulfill the inequality 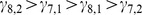, see Table 1. Equations (1) and (4) thus imply that the impact of the *At*GRP8 protein on the alternative splicing of its own (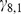) and of the *AtGRP7* pre-mRNA (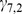) is weaker than that of the *At*GRP7 protein on the alternative splicing of its own (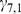) and of the *AtGRP8* pre-mRNA (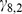). This suggests that *At*GRP8 oscillations are subordinated to those of *At*GRP7. Upon adopting for *At*GRP8 the same parameters as for *At*GRP7 (see Table 1), apart from the constants connected to alternative splicing (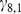 and 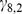) and transcription kinetics (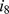, 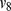, and 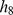), the mRNA oscillations still behave qualitatively similar (see Figure S4) and the earlier peak of *AtGRP8* mRNA persists. Altogether this suggests that the higher impact of *At*GRP7 on the alternative splicing could be the essential mechanism underlying the earlier *AtGRP8* mRNA peak compared to that of *AtGRP7* mRNA.

#### Our model suggests highly saturated protein degradation

Our analysis revealed very low activation coefficients 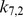 and 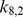 of the corresponding protein degradation kinetics (see Table 1). As a consequence, the corresponding protein dynamics (right hand side of equations (3) and (6)) exhibits a notable dependence on the protein concentrations 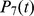 and 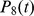 themselves only if these concentrations are extremely small (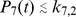 and 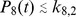). The resulting straight decay in *At*GRP7 protein concentration to a value close to zero and the concomitant suspension of negative auto-regulation via alternative splicing leads to the observed fast recovery of pre-mRNA concentrations 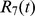 and 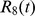 from their trough values (see Figure 2 and equations (1) and (4)). For protein concentrations much larger than the activation coefficients (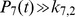 and 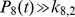) the dynamics in equations (3) and (6) are solely governed by the mRNA concentrations 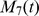 and 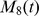. In other words the degradation kinetics of *At*GRP7 and *At*GRP8 proteins are highly saturated.

#### Our model accounts for LHY-ox, *ztl*, and *toc1* mutant data

Introducing LHY/CCA1 as a transcriptional repressor of *AtGRP7* and *AtGRP8* was motivated by experiments, showing that *AtGRP7* mRNA oscillations are damped to their trough value in plants constitutively over-expressing CCA1 [26] or LHY [27] under LL conditions (see *Introduction*). We simulated the LHY or CCA1 over-expression plants by adding a constant, unregulated transcription rate 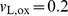 to the differential equation of the *LHY/CCA1* mRNA in the core oscillator model by *Pokhilko et al.* [11]. The resulting time traces of the LHY overexpression mutant indeed show the experimentally observed damping of *AtGRP7* mRNA to the trough value of its corresponding wild type oscillations under LL conditions, see Figure S5 A.

Plants carrying a mutation in the gene of the F-Box protein ZEITLUPE (ZTL) were shown to exhibit *AtGRP7* mRNA oscillations [45] and *CCR2*::LUC (*AtGRP7*::LUC) expression [46] with a markedly prolonged period under free-running conditions. This behavior is also visible in our model (see Figure S5 B), where we simulated the *ztl* null mutant by setting the production of ZTL to zero: Under LL conditions, this mutant shows self-sustained LHY/CCA1 oscillations with an approximately 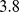
h longer period compared to the 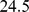
h wild type behavior, which in turn entrain *At*GRP7 to this rhythmicity.

Similarly, a hypothetical clock mutant (as described in [11]), neglecting the transcriptional repression of *PRR9* by TOC1 accounts for the experimentally observed short period of *AtGRP7* mRNA oscillations in *toc1* mutant plants under LL conditions [47], see Figure S5 C. This is again meditated through the experimentally observed reduced period of *LHY/CCA1* oscillations [48]. Note that the simulated *toc1* null mutant, realized in the model from [11] by setting the production of *TOC1* mRNA to zero, shows stronger damping and an unrealistically strong phase shift in LL, but still retains the experimentally observed period shortening, see Figure S5 D.

### The Slave Oscillator Can Be Viewed as a Driven Bistable Toggle Switch

Two genes that mutually repress each other by transcriptional inhibition are known to constitute a genetic *toggle switch* – a prototypical example of a biological system showing bistability [49]. *Gardner et al.* reconstructed such a toggle switch in *Escherichia coli* and proposed a two variable model (*Gardner model*) in order to explain the necessary conditions for bistability [50]. In the system studied here, both genes, *At*GRP7 and *At*GRP8, also cross-regulate each other. However, the reciprocal regulation of *At*GRP7 and *At*GRP8 occurs at the post-transcriptional level via alternative splicing followed by nonsense-mediated decay of the alternative splice forms instead of mutual inhibition of transcription. This led us to the question whether the slave oscillator could act as a toggle switch. Therefore, we decoupled the slave oscillator from the core oscillator by setting 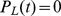 for all times 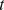, thus neglecting the transcriptional repression of *AtGRP7* and *AtGRP8* by LHY/CCA1. In other words *AtGRP7* and *AtGRP8* are now transcribed at constant rates 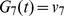 and 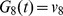, respectively, see also text below equation (6). Note that both *At*GRP7 and *At*GRP8 show negative auto-regulation as an additional feature not described for the toggle switch as proposed in [50].

As a first step, we investigated whether our decoupled slave oscillator system (i.e. equations (1)–(6) with 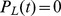) can exhibit bistability. While we show in Text S1 B that the simplified model with a single *At*GRP7 feedback loop can only have one fixed point (either stable or unstable), the interlocked *At*GRP7 and *At*GRP8 feedback loop may give rise to bistability, i.e. a scenario where two stable steady states can coexist: In order to test the system's ability to show a bistable behavior, we randomly sampled parameter sets in the same range as before. A linear stability analysis applied to every fixed point of a given parameter set (see *Methods*) revealed a monostable longterm behavior in 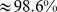 of all cases, bistability in 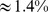, and oscillatory behavior in 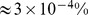. Such oscillations were not possible in the two-variable model by *Gardner et al.* [50]. Moreover, we found that a tiny rest of about 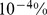 exhibited still other phase space structures, such as the coexistence of a stable fixed point and a limit cycle attractor.

**Figure 4:**
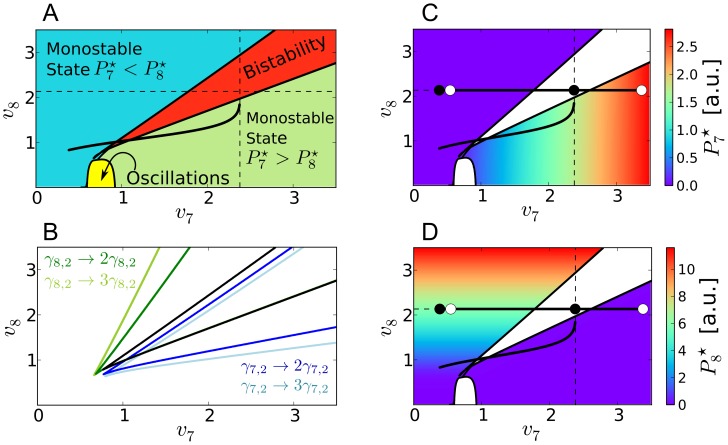
The slave oscillator may represent a driven bistable toggle switch. A) The 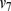
–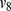-bifurcation diagram of the slave oscillator decoupled from the core oscillator consists of four main regions: two monostable areas (*blue* and *green*), a bistable area (*red*), and an area where autonomous oscillations are possible (*yellow*). Dashed lines indicate the directions in parameter space used for the one parameter bifurcation diagrams in Figure S6. The intersection of these lines marks the optimal parameter set from Table 1. The black curve is discussed in detail in the main text. B) Modification of the splicing coefficients 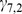 and 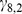, responsible for the reciprocal cross-regulation, affects the slope of the boundaries between the bistable and the monostable regions (*black*: original boundaries, *color*: modified boundaries). C) & D) Color-coded fixed point concentrations 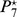 and 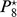 of *At*GRP7 and *At*GRP8 protein in the monostable areas. Straight lines with black and white dots are explained in the main text.

Figure 4 A illustrates the situation when only the two parameters 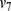 and 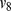 are varied, while all other parameters are kept at their values from Table 1. Such variations of 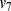 and 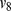 are of particular interest since they effectively correspond to variations of 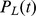 at fixed 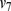 and 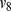 in equations (1) and (4): The transcription of *AtGRP7* and *AtGRP8* is repressed whenever 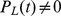 and the corresponding transcription rates 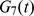 and 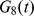 (see text below equation (6)) adopt values smaller than their maximal transcription rates 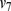 and 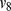.

For our optimal parameter set from Table 1, the system shows bistability (see intersection of the dashed lines in Figure 4 A). Similar to the *Gardner model* [50] a bistable region separates two monostable regimes in Figure 4 A. In those two monostable regions either high *At*GRP7 fixed point protein concentrations 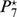 dominate over *At*GRP8 fixed point protein concentrations 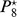 or *vice versa* (Figures 4 C and D). The one parameter bifurcation diagrams, following the dashed lines in Figures 4 A, show the typical hysteretic behavior of a toggle switch (Figures S6). Intuitively understandable, the 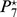 and 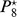 protein fixed point concentrations increase with increasing maximal transcription rates 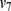 and 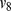, respectively.

In the *Gardner model* [50] the degree of cooperativity of the reciprocal transcriptional inhibition determined the slope of the bifurcation lines and therefore the size of the bistable region. In our case, the strength of the reciprocal control of alternative splicing (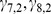) has an analogous effect, as one can see in Figure 4 B. An increase of the splicing coefficient 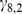 nearly exclusively alters the slope of the bifurcation line bordering the monostable region where *At*GRP8 protein dominates, and similarly for 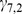.

*Gonze* already showed in 2010 that periodically forcing the transcription of one of the two genes in the *Gardner model* can induce limit cycle oscillations [51]. Specifically, high forcing amplitudes can drive the system from one monostable region to the other by crossing the bistable regime. In our system, the LHY/CCA1 protein 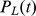 in equations (1) and (4) was assumed to affect both *AtGRP7* and *AtGRP8* transcription. We therefore have to investigate this phenomenon in a two parameter bifurcation diagram. Indeed, if we pursue the trajectory of the transcriptional rates 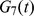 and 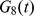 (see text below equation (6)) of *AtGRP7* and *AtGRP8* (black curved line in Figure 4 A) during one cycle under 12h∶12h LD conditions, one observes that the rhythmic transcriptional repression via LHY/CCA1 drives the system from one monostable region to the other by crossing a narrow bistable branch. This is only possible due to different kinetics of *AtGRP7* and *AtGRP8* transcription (see Table 1). Completely identical transcription kinetics for *AtGRP7* and *AtGRP8* (i.e. 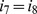, 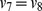, 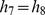, and therefore 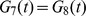) would lead to a straight line of unit slope in the 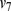
–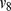 bifurcation diagram instead of the curved shape, not allowing the system to reach one monostable region from the other.

#### Neglecting the transcriptional repression of *AtGRP8* by LHY/CCA1 protein

**Figure 5:**
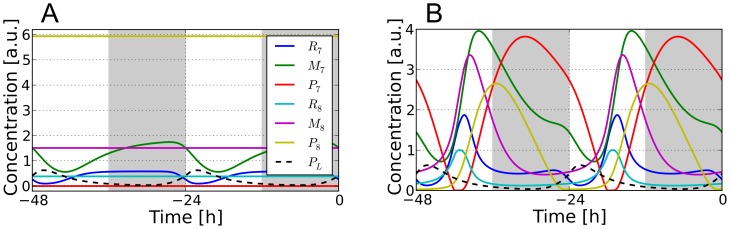
Stable oscillations can be observed even without transcriptional repression of *AtGRP8*. A) Solutions of equations (1)–(6) for the “optimal” parameter set from Table 1 after neglecting the repression of *AtGRP8* transcription by LHY/CCA1, i.e. 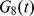 is held constant at the value 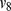 from Table 1. B) Same as in A) after additionally increasing the maximal transcription rate 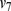 to 3.38. Shown are the last two days in 12h∶12h LD conditions (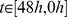).

In our model we have assumed a transcriptional repression of *AtGRP8* by LHY/CCA1 for reasons of similarity with *AtGRP7*. Since this was never investigated experimentally so far, we asked whether it would be possible to still reproduce the experimental findings without this hypothetical repression. In the present framework, this is tantamount to keeping 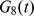 constant at the value 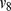. As a result, the system moves back and forth between the black dots in Figure 4 C and D without fully crossing the bistable region. Hence we can conclude that 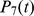 remains at an almost constant low value and 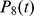 at an almost constant high value. Figure 5 A confirms this expected behavior together with a similar behavior of 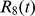 and 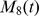, while 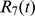 and 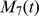 still exhibit appreciable oscillations (which in turn could be expected from Figures S7 A and B). In other words, we obtain a strong disagreement with the known experimental facts 1–5 (see section *Parameter Estimation*).

However, this problem can be readily solved by increasing the maximal transcription rate 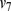, e.g. from 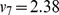 to 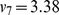, so that the system now moves back and forth between the white dots in Figure 4 C and D, and in particular fully crosses the bistable region. As a result, an oscillatory behavior of 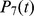 and 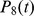 is recovered similar to the original oscillations in Figure 2 and likewise for the other concentrations, see Figure 5 B. The main difference is the somewhat higher maximum of the 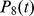 oscillations, in qualitative agreement with Figure 4 D.

#### Impact of saturated protein degradation on the bifurcation diagrams

For the optimal parameter set (see Table 1) the protein degradation is highly saturated (i.e. the activation coefficients 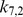 and 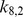 are very small, see also end of section *Comparison with Experimental Results and Computational Predictions*). Consequently, the fixed point concentration in equation (20) can be approximated as 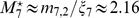 if 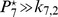. In view of the bifurcation diagram in Figure 4 C this explains the region uniformly colored red in Figure S7 B, i.e. the fixed point concentration 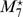 is nearly constant and equal to 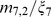 in the whole area of the 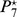 dominated monostable area. According to equation (21), the pre-mRNA fixed point concentration 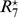 also remains almost constant (see red region in Figure S7 A). Analogous considerations apply to 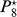, 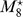, and 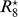.

This behavior could also be of theoretical interest since a highly saturated degradation kinetics allows the system to change the value of one variable (here the protein concentration 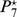 or 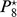) while keeping all other fixed point concentrations constant. This would imply yet another potential function of a saturation kinetics in addition to its recently discussed role as an efficient mechanism of inducing delay into negative feedback loops in order to favor oscillations [52]–[54].

### Robustness against Variations in the LHY/CCA1 Protein Oscillations

**Figure 6:**
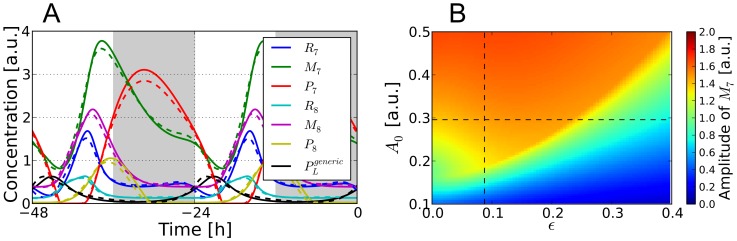
Systems dynamics driven by a modified Poincaré oscillator. A) *Dashed*: Same traces as shown for LD conditions in Figure 2. *Solid*: Same but with a core oscillator input 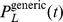 generated by a modified Poincaré oscillator with parameters 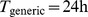, 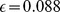, 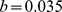, and 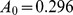, as detailed in section *Methods*. B) Amplitude of the 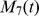 oscillations when the slave oscillator is driven by a generic Poincaré oscillator of different amplitudes 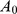 and waveform parameters 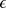 at fixed 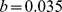 and 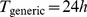. The point of intersection of the dashed curves indicates the parameters 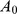 and 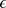 used in A).

In order to examine the effect of variations in the core oscillator input 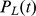 on the *At*GRP7-*At*GRP8 slave oscillator we substituted the core oscillator protein concentrations 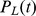 obtained from the model of *Pokhilko et al.* [11] by 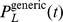 obtained from a modified Poincaré oscillator, similar to the model used in [55]. This generic oscillator, described in detail in the section *Methods*, is tunable in its period 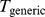 and amplitude 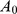. A third parameter 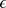 determines the shape of the oscillations, ranging from sinusoidal (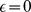) to increasingly spiky oscillations with increasing 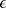, and a fourth parameter 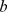 determines the trough value. In particular, for 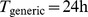, 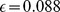, 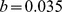, and 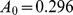, the resulting oscillations 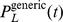 are very similar to 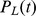 under 12h∶12h LD conditions (see black lines in Figure 6 A). Likewise, the corresponding slave oscillator dynamics differ only little from those obtained by a coupling of the *At*GRP7-*At*GRP8 feedback loops to the more complex core oscillator model [11], as one can see in Figure 6 A. In other words, we can replace the complex core oscillator model, being composed of many differential equations and parameters, by any other model which faithfully imitates the actual protein oscillations of LHY/CCA1.

In particular, we verified that almost identical solutions for the slave oscillator dynamics are recovered (exemplified for 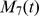 by Figure S8), when we replace our original model from [11] by the recently published refined core oscillator model from [13]. While shape and phase of the LHY/CCA1 protein oscillations are fairly similar in both core oscillator models, the amplitude of 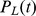 approximately doubles for the refined model from [13]. As expected from equations (1) and (4), adapting the activation coefficients according to 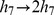 and 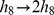 then results in almost identical results for the slave oscillator, see Figure S8.

It is known that oscillations, governed by a hysteretic switch mechanism, exhibit oscillations with a robust amplitude, mainly determined by the height of the hysteretic loop, while being easily tunable in their period [56], [57]. In order to investigate the effect of changes in the LHY/CCA1 protein concentrations 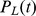, and whether our driven *At*GRP7-*At*GRP8 slave oscillator shows robust amplitudes for varying 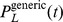 as well, we examined the behavior of the system for different amplitudes 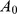 and waveforms 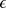 of the core oscillator while keeping 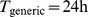 and 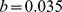 constant. Figure 6 B shows the color-coded values of 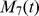 amplitude obtained from simulations with different 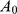 and 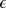. For a given shape parameter 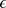, the amplitude of 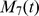 oscillations nearly stays constant after reaching a certain driving amplitude 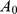 even if we further increase 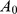, i.e. the values of 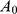 are strong enough to overcome the bistable region and to repress the system to a trough value of the 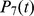 oscillations close to zero. This threshold amplitude increases for more spiky oscillations with increasing 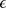 since the timespan of the transcriptional repression becomes shorter and the systems dynamics needs time to react to the corresponding “movement” in the 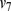
–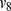 bifurcation diagram in Figure 4 A (similar diagrams can be obtained for the other concentration species 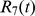, 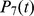, 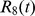, 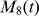, and 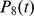). Nevertheless, oscillations of robust amplitudes can be induced for a wide range of combinations of 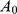 and 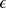 (see red area in Figure 6 B).

### Limitations of the Model: The *lhy cca1* Double Mutant

**Figure 7:**
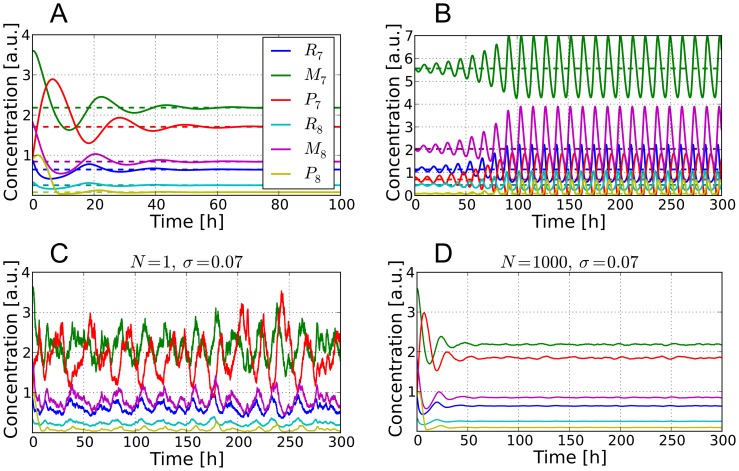
Damped, autonomous, and noise-induced oscillations after decoupling the slave from the core oscillator. A) Relaxation dynamics are observed for the optimal parameter set from Table 1. Dashed lines denote the corresponding fixed points. B) After changing the *At*GRP7 and *At*GRP8 protein degradation rates to 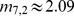 and 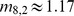, respectively, the slave oscillator develops autonomous oscillations. C) Pure noise-induced oscillations of a single cell (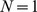) for the parameter set from Table 1. D) Same after averaging over an ensemble of 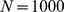 cells. See Text S1 C/D for further details (especially the noise-strength 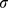).

The *lhy cca1* double mutant does not express LHY and CCA1, hence the protein concentration 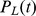 of the core oscillator must vanish. We have shown in the previous section that the resulting autonomous dynamical system (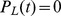 in equations (1)–(6)) approaches a steady state in the bistable region, see Figures 4 A and 7 A, i.e. oscillatory solutions are ruled out. This theoretical result is in contradiction to the experimental finding that the *AtGRP7* transcript shows diurnal oscillations with a phase shift to dawn in the *lhy cca1* double mutant [58]–[60].

As a first possible resolution of this contradiction we considered the possibility of modifying the kinetic parameters of Table 1 without changing our model (1)–(6) itself in order to generate oscillatory solutions of the autonomous dynamics (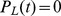). As demonstrated by Figure 7 B and detailed in Text S1 C this is indeed possible but the obtained periods of oscillation are prohibitively small. Moreover, tiny parameter variations in an ensemble of autonomous oscillators will lead to deviating oscillation periods and hence the oscillations average out in the longterm.

Next we considered the possibility to explain the experimental facts by means of noise effects. Indeed, noise is omnipresent in biological systems due to the probabilistic nature of molecular reactions or fluctuating environmental influences [61], [62] and noise induced oscillations have been reported in numerous other models [63]–[65]. Again, as shown in Figures 7 C/D and detailed in Text S1 D, we were able to generate noise-induced self-sustained oscillations on the single cell level, but not in the ensemble.

An obvious remedy in both our attempts discussed above is to introduce coupling between the individual oscillators. However, in the experimentally relevant case of many cells the details of their mutual interaction are still not fully clarified, but a global synchronization mechanism seems unlikely [66]–[68]. Moreover, we note that both our attempts are also unable to explain one more experimental fact, namely the entrainment of *AtGRP7* mRNA oscillations to 24h-periodic light-dark cycles in the *lhy cca1* double mutant [59], [60]. In conclusion, the only remaining possibility to explain the observed rhythmicity of *AtGRP7* mRNA in *lhy cca1* double mutants seems to include to the model (1)–(6) additional influences of the core oscillator variables (as already stated, a direct influence of light seems negligible (unpublished data)), e.g. additional transcriptional activators or inhibitors.

### Conclusion

We introduced and analyzed a mathematical model for the molecular regulatory network of the *At*GRP7 and *At*GRP8 slave oscillator in *Arabidopsis thaliana*. Based on experimental results, we assumed that the slave oscillator gains input from the circadian core oscillator via transcriptional repression by the LHY/CCA1 proteins. Furthermore, we assumed that it shapes its oscillatory profile due to a negative auto-regulation and reciprocal cross-regulation between *At*GRP7 and *At*GRP8 via alternative splicing followed by nonsense-mediated decay of the alternative splice form. Although alternative splicing is abundant among circadian clock genes [69], [70], this is as far as we know the first mathematical model of a circadian clock-related molecular network that includes alternative splicing as a regulatory mechanism. We determined the model's kinetic parameters by a two-step optimization process including random sampling and an evolutionary algorithm. With the resulting optimal parameter set we could successfully reproduce most of the pertinent experimental findings such as waveforms, phases, and half-lives of the time-dependent concentrations. Furthermore, the model can account for experimentally observed mutant behavior in LHY-ox, *ztl*, and *toc1* mutant plants. The observed *AtGRP7* mRNA oscillations can be sufficiently explained through the altered behavior of the LHY/CCA1 protein oscillations in these mutants.

We note again that the slave oscillator, since it is driven by the core oscillator, does not have to share all the properties of the core oscillator such as self-sustaining oscillations, temperature compensation, or direct light input [19], [20]. Indeed, we find dampened dynamics rather than independent self-sustained oscillations for the optimal parameter set from Table 1 (see e.g. Figure 7 A).

The model can also be used to predict properties not considered by our optimization procedure or properties not measured so far. It suggests a shorter half-life of *AtGRP8* compared to *AtGRP7* mRNA and a fast and highly saturated protein degradation of both *At*GRP7 and *At*GRP8. The latter finding is consistent with recent experimental results showing that *At*GRP7 and *At*GRP8 proteins are among those with the highest degradation rates in *Arabidopsis thaliana* [71]. Furthermore, the model revealed that *At*GRP7 may have a stronger impact on the alternative splicing of the *AtGRP7* and *AtGRP8* pre-mRNAs than *At*GRP8. This may be the mechanism underlying the observed earlier peak of *AtGRP8* mRNA compared to *AtGRP7* mRNA.

As highlighted in [72] it might also be interesting to investigate the persistence of the above general predictions for parameters which differ from the optimal parameter set considered so far. Figure S9 indicates that the subordination of *AtGRP8* to *AtGRP7* seems to be a robust feature of the optimization procedure, while the other two features (shorter *AtGRP8* mRNA half-life and saturated protein degradation) seem to be less robust.

Our modeling process also provided theoretical insight into possible mechanisms underlying the experimentally observed *At*GRP7 and *At*GRP8 oscillations: The slave oscillator model from equations (1)–(6) is potentially able to show bistability and indeed does so for the parameter set found by our optimization scheme, suggesting that the core oscillator basically triggers periodic switching of the slave oscillator between two monostable branches by crossing a bistable regime. Our *At*GRP7-*At*GRP8 slave oscillator could therefore be the first *in vivo* manifestation of the purely theoretical proposal of a genetic toggle switch driven by an autonomous self-sustained oscillator [51].

What evolutionary benefit could such a mechanism have? It is known that oscillations based on a hysteretic switch can show robust amplitudes. Indeed, our present slave oscillator also shows oscillations which are robust in amplitude for a considerable variety of different driving oscillations 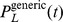. The formation of a driven interlocked auto-regulatory feedback loop that originated from a gene duplication event in the case of *At*GRP7 and *At*GRP8, can thus lead to a system showing a hysteretic behavior and resulting, if forced with an appropriate amplitude, in oscillations with a robust amplitude.

Finally, we proposed two possible changes in the current view of the regulatory network of *At*GRP7 and *At*GRP8:

First, we can still reproduce the experimental findings even without the common assumption of transcriptional repression of *AtGRP8* by LHY/CCA1. Up to now, the latter assumption has been justified by reasons of similarity with *At*GRP7 but not by direct experimental measurements [26], [27].

Second, we have discussed modifications of the model (1)–(6) in order to reproduce the experimental behavior in the *lhy cca1* double mutant. In contrast to the simulation of the *lhy cca1* double mutant, the *AtGRP7* transcript shows oscillatory behavior with a phase shift to dawn under entrainment conditions [58]–[60]. We therefore tested natural possibilities how to cure this shortcoming of the model: Two of them, namely the autonomous oscillations due to noise effects and a change of the kinetic parameters from Table 1 could be readily excluded since they cannot explain the phase locking of the *AtGRP7* mRNA in the *lhy cca1* double mutant to 24h-periodic light-dark cycles. We therefore concluded that additional influences of the core on the slave oscillator, on top of the transcriptional repression by LHY/CCA1, have to be incorporated to consistently explain both the wild-type *and* the *lhy cca1* double mutant behavior.

Furthermore, it has to be taken into account that *At*GRP7 influences many physiological processes: It promotes the floral transition at least partly by down-regulating the floral repressor *FLC* [33]. Furthermore, it plays a role in the plants innate immune system since *grp7-1* plants that do not produce *At*GRP7 mRNA are more susceptible to *Pseudomonas syringae* [34], [35]. *At*GRP7 is also known to mediate responses to stresses such as oxidative stress, high salt, mannitol, or cold [21], [36], [37]. Our modeling results could be used in future work to integrate the *At*GRP7 and *At*GRP8 feedback loops with these other regulatory cues.

## Methods

### Equation Solving

The numerical solutions of equations (1)–(6), or equivalently of equation (7), have been obtained by using the *odeint* function of *SCIentificPYthon* which uses LSODA from the Fortran library ODEPACK. In particular, we remark that LSODA is able to identify and solve initial value problems for both stiff and non-stiff problems.

### Cost Function and Parameter Estimation

In this section we provide the details of the optimization procedure as referred to in the section *Parameter Estimation*. Similarly as in [7], we started our search for an optimal fit by generating 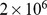 *Antonov-Saleev* quasi-random parameter sequences 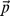 (adopting the *gsl_qrng_sobol* routine from the GNU Scientific Library) that were subsequently tested for their fitness 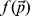 (for the explicit definition of 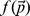, see Text S1 A). To take into account the similarity of *At*GRP7 and *At*GRP8 we first sampled the parameters for a reduced system consisting only of *At*GRP7, see also Text S1 B. After this random sampling step, the network motif was extended to the complete system while choosing the parameters in order to generate two identical oscillatory profiles for *At*GRP7 and *At*GRP8 (upon comparison of equation (1)–(6) in the main text and those in Text S1 B, all parameters have to be duplicated except for the rate constant 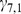 which has to be set to the half of its previous value and then has to be identified with 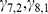 and 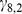).

In the next step, we took the best one hundred 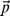 values and further minimized the cost function 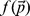 in their local neighborhood. In order to solve this 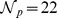 dimensional minimization problem we used the gradient-free Nelder Mead Downhill Simplex method, where an initial simplex with 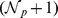 vertices, including the starting parameter set, “crawls” amoeba-like via shape transformations (*reflection*, *contraction* and *expansion*) through parameter space in the direction of lower cost 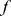 [41]. We modified the original algorithm in a way that negative and therefore biologically not meaningful parameter values were penalized by setting the cost-function value of such vertices to infinity. The starting simplex was defined by the initial parameter set 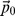 and the set of vertices defined by 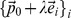 where the 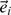
's are the 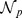 unit vectors in each parameter space's direction and 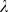 is a constant chosen to be 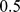 in our simulations. The reflection, expansion and contraction coefficients 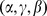 were chosen as 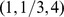 throughout the simulations and after the algorithm claimed to be finished it was restarted four times from the best point found in the previous run.

We also tried out a Monte-Carlo Hillclimbing method instead of the simplex optimization, which however led to worse results.

### Fixed Points, the Jacobian, and Bifurcation Analysis

As detailed in the main text, the *lhy cca1* double mutant can be modeled by setting 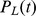 in equations (1) and (4) to zero for all times 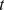. In the slave oscillator model proposed here, this is equivalent to the deletion of all links to the core oscillator. Equations (1)–(6), or equivalently (7), then define an autonomous dynamical system which is easy enough to calculate the fixed points 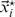 analytically.

More precisely, for one component of the fixed point 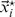, namely 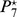, one obtains the following closed quartic equation
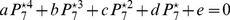
(9)with coefficients
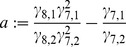
(10)

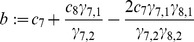
(11)

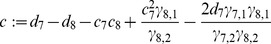
(12)

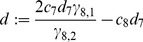
(13)

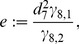
(14)and abbreviations
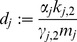
(15)

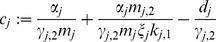
(16)

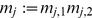
(17)

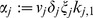
(18)for 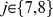.

In principle the quartic equation (9) can be solved analytically by means of the formula of *Cadano & Ferrari*. We used the root finding package *root* of *SCIentificPYthon* instead. In general, we thus obtained four different solutions 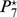 of the quartic equation (9).

Once these four solutions 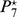 are determined, the remaining components of the four fixed points 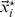, 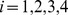, can be readily obtained from the equations
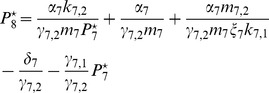
(19)

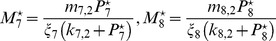
(20)

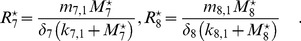
(21)


For the optimal parameter set from Table 1 we thus obtained the following four fixed points
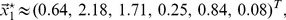
(22)

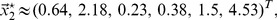
(23)

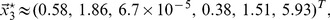
(24)

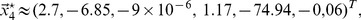
(25)where the last one is not biologically meaningful due to its negative concentration values.

A standard linear stability analysis based on the eigenvalues of the Jacobian matrix
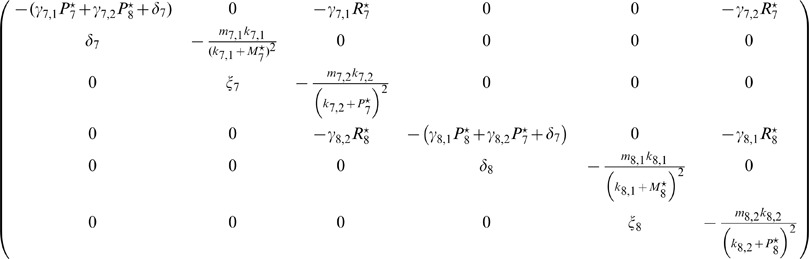
(26)reveals that two of the remaining fixed points (22),(23), and (24) are (locally) stable (namely 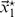 and 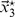) and one is (locally) unstable (namely 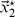).

A similar algorithm was used to generate Figure 4 and Figures S6 and S7: For each parameter set 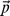 we first calculated the four fixed points as described above. In a next step, those with negative or complex components were sorted out. Finally, we performed a linear stability analysis as described above.

### Tunable Modified Poincaré Oscillator

In order to better highlight the dependence of our slave oscillator on properties like the amplitude or peak broadness of 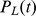, we replaced the differential equations for the molecular core oscillator model provided by *Pokhilko et al.* [11] by an easily tunable generic oscillator in the form of a modified nonuniform *Poincaré oscillator* as proposed in [55]. Its radial evolution is given by
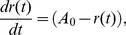
(27)therefore converging for any initial condition 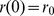 to the stable fixed point 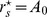, amounting to the amplitude of the resulting oscillations. The phase dynamics are given by
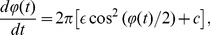
(28)where 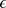 determines the shape of the oscillations, ranging from a sinusoidal (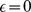) to a more and more spiky oscillator (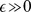) with period
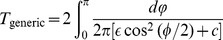
(29)

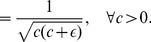
(30)


Note that the period depends on the choice of both parameters 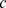 and 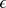. In [55], the model parameter 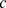 in (28) was originally chosen as a small non-zero positive constant in order to make sure that 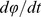 never becomes zero, since for 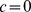 the solution of equation (28) would evolve to its fixed point in phase 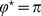. For our purpose, we set
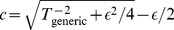
(31)so that, for any given 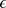, the oscillator exhibits oscillations with a fixed period 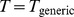. Finally, we define
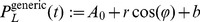
(32)as the input substituting the LHY/CCA1 oscillations 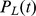 in (1) and (4). The extra parameter 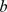 in (32) denotes the trough value of the oscillations and is set to the trough-value 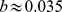 of the 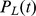 oscillations.

## Supporting Information

Figure S1 Simulations of the “best” parameter set, obtained via the *full* parameter space sampling-procedure, fit the experimental time traces worse (for a comparison, see Figure 3 A). *Blue*: Simulated *AtGRP7* mRNA oscillations. *Green*: “COL_LDHH” experimental data set from the DIURNAL database, as used for Figure 3 A. The time traces were normalized to their maximal expression values, defined as 1.
(TIFF)

Figure S2A gradual decrease of the (alternative) splicing coefficient 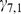, which accounts for the negative auto-regulation of *At*GRP7, shifts the phases of the *AtGRP7* mRNA oscillations (A) and of the pre-mRNA oscillations (B) to a later time of day. On top of that, the peaks of the oscillations get increasingly broader.(TIFF)

Figure S3A) & B) *In silico* half-life experiments for *AtGRP7* (A) and *AtGRP8* (B) mRNA following an experimental protocol (see main text and Text S1 A). In our model, the mRNA and protein half-lives were shown to depend on the day-time at which transcription or translation were stopped, respectively. The dark-blue and red lines denote the same *AtGRP7* and *AtGRP8* mRNA traces as shown in Figure 2. The light-blue and orange lines denote the dynamics after the interruption of transcription. C/D/E/F) Represented are the resulting half-lives 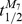, 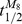, 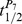, and 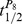 over a full diurnal cycle (blue lines) for *AtGRP7* (C) and *AtGRP8* (D) mRNA as well as *At*GRP7 (E) and *At*GRP8 (F) protein, respectively. Dashed green lines denote the same *AtGRP7* mRNA (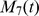), *AtGRP8* mRNA (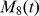), *At*GRP7 protein (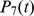), and *At*GRP8 protein (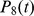) concentrations as in Figure 2. All figures were obtained under 12h∶12h LD conditions.(TIFF)

Figure S4Dashed: Simulations for the optimal parameter set from Table 1, identical to those of Figure 2. Solid: Even if one adopts for *At*GRP8 the same parameters as for *At*GRP7 (see Table 1), apart from the constants connected to alternative splicing (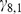 and 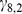) and transcription kinetics (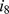, 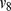, and 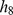), the mRNA oscillations of *AtGRP7* and *AtGRP8* still behave qualitatively similar. In particular, the earlier peak of the *AtGRP8* mRNA persists.(TIFF)

Figure S5 Simulations of the LHY overexpression (LHY-ox) mutant (A), *ztl* (B), and *toc1* (D) null mutants as well as a hypothetical *toc1* mutant (C), where the repression of PRR9 by TOC1 is neglected, as described in [11]. *Dashed lines* denote the wild type (wt) and *continuous lines* denote the mutant simulations of *AtGRP7* mRNA (*green*) and LHY/CCA1 protein oscillations (*black*).
(TIFF)

Figure S6One parameter bifurcation diagrams of the maximal transcription rates 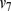 (*left*) and 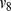 (*right*), corresponding to the dashed lines in Figure 4 A/C/D. The protein concentration values 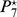 and 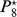 for stable fixed points are plotted in red and blue, respectively. Protein concentrations for unstable fixed points are kept in black. Dashed lines indicate the parameter values from the optimal parameter set of Table 1.(TIFF)

Figure S7Analogously to Figures 4 C/D of the main text, we plotted the color-coded fixed point concentrations of the *AtGRP7* pre-mRNA (A) and mRNA (B) as well as the *AtGRP8* pre-mRNA (C) and mRNA (D) in the monostable areas of the 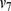
–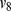 bifurcation diagram. The intersection of the dashed lines marks the optimal parameter set from Table 1.(TIFF)

Figure S8Solid: Reproduction of the results for 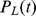 and 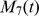 from Figure 2. Dashed: Corresponding results after replacing the original core oscillator model from [11] by the refined model from [13] and adapting the activation coefficients according to 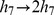 and 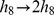.(TIFF)

Figure S9Four key features of the model dynamics (1)–(6) under 12h∶12h LD conditions for the optimal parameter set from Table 1 (Ranking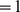) and for the 19 next best parameter sets (Ranking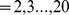) resulting from the above described two-step optimization process with random initialization and subsequent evolutionary optimization. A) Two representative examples of the 20 (sub-)optimal parameter sets (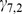 and 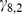). As detailed in the main text, the observed general property 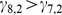 indicates that the subordination of *At*GRP8 to *At*GRP7 is a robust feature of our optimization procedure. The experimentally observed earlier peak of *AtGRP8* mRNA compared to *AtGRP7* mRNA, i.e. 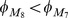 (see section *In silico waveforms and phases are consistent with the experimental data*), is a further such robust feature. B) The half-lives 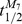 and 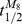 (see main text and Text S1 A) indicating that the shorter life-time of *AtGRP8* mRNA compared to *AtGRP7* mRNA is a less robust feature of our optimization procedure. Likewise, the depicted Michaelis constants 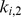 and the peak (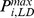) and trough values (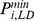) of 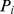 oscillations in C) (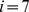) and D) (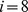) indicate that the saturation of *At*GRP7 and *At*GRP8 protein degradation is a less robust feature.(TIFF)

Figure S10Function 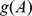 (see Text S1 A) is plotted versus different peak-trough-values 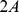. The peak-trough-values 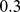 and 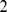, each leading to a cost function contribution 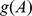 of one, are indicated by vertical dashed lines.(TIFF)

Text S1 A) Detailed description of the cost function. B) Analysis of the one-component posttranscriptional feedback loop. C) Search for self-sustained oscillations. D) Search for noise-induced oscillations.
(PDF)
