# Supplementary figures and images for: A Circadian Clock-Regulated Toggle Switch Explains AtGRP7 and AtGRP8 Oscillations in Arabidopsis thaliana
Source: PLoS Comput Biol. 2013 Mar 28;9(3):e1002986. doi: 10.1371/journal.pcbi.1002986 (PMC3610657; doi:10.1371/journal.pcbi.1002986)

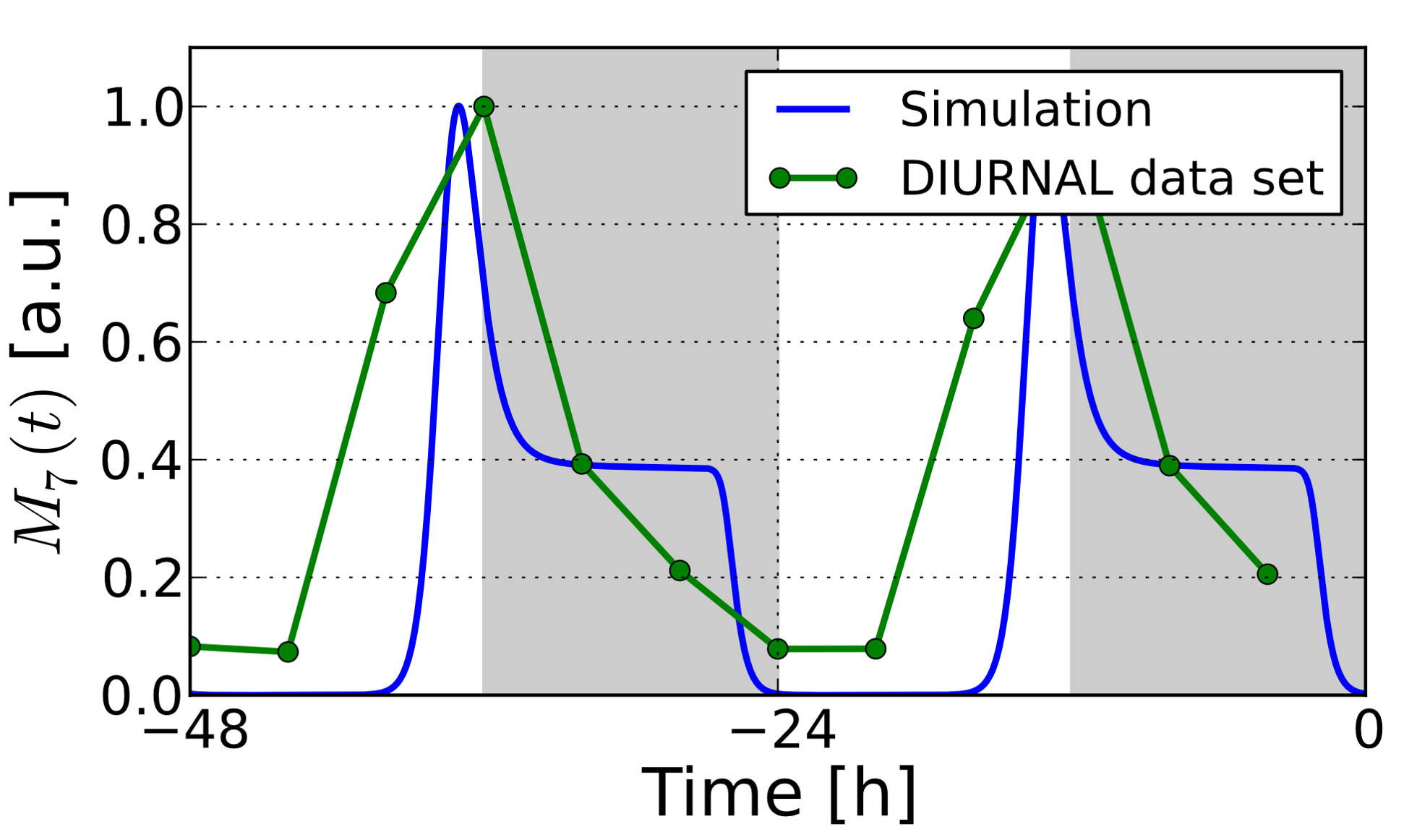

Supplement: Figure S1 — Simulations of the “best” parameter set, obtained via the full parameter space sampling-procedure, fit the experimental time traces worse (for a comparison, see Figure 3 A). Blue: Simulated AtGRP7 mRNA oscillations. Green: “COL_LDHH” experimental data set from the DIURNAL database, as used for Figure 3 A. The time traces were normalized to their maximal expression values, defined as 1. (TIFF) [file pcbi.1002986.s001.tif]

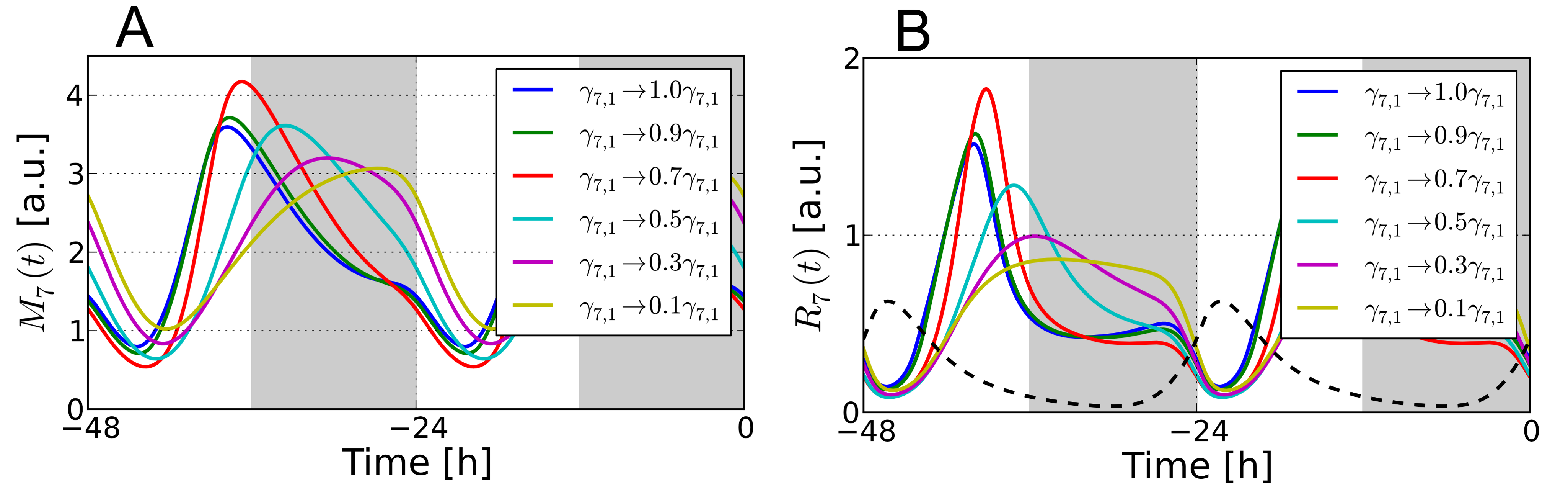

Supplement: Figure S2 — A gradual decrease of the (alternative) splicing coefficient , which accounts for the negative auto-regulation of AtGRP7, shifts the phases of the AtGRP7 mRNA oscillations (A) and of the pre-mRNA oscillations (B) to a later time of day. On top of that, the peaks of the oscillations get increasingly broader. (TIFF) [file pcbi.1002986.s002.tif]

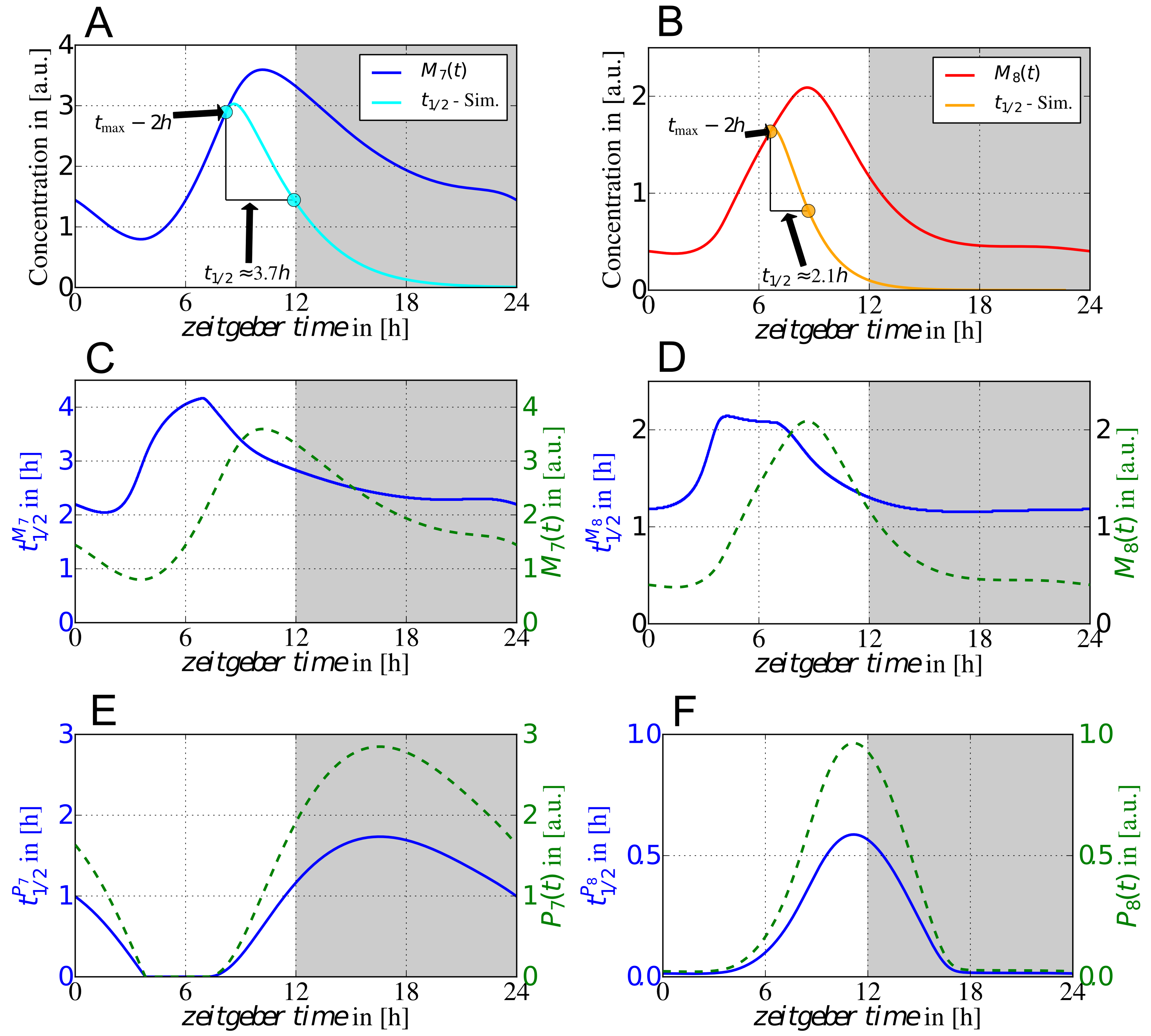

Supplement: Figure S3 — A) & B) In silico half-life experiments for AtGRP7 (A) and AtGRP8 (B) mRNA following an experimental protocol (see main text and Text S1 A). In our model, the mRNA and protein half-lives were shown to depend on the day-time at which transcription or translation were stopped, respectively. The dark-blue and red lines denote the same AtGRP7 and AtGRP8 mRNA traces as shown in Figure 2. The light-blue and orange lines denote the dynamics after the interruption of transcription. C/D/E/F) Represented are the resulting half-lives , , , and over a full diurnal cycle (blue lines) for AtGRP7 (C) and AtGRP8 (D) mRNA as well as AtGRP7 (E) and AtGRP8 (F) protein, respectively. Dashed green lines denote the same AtGRP7 mRNA (), AtGRP8 mRNA (), AtGRP7 protein (), and AtGRP8 protein () concentrations as in Figure 2. All figures were obtained under 12h∶12h LD conditions. (TIFF) [file pcbi.1002986.s003.tif]

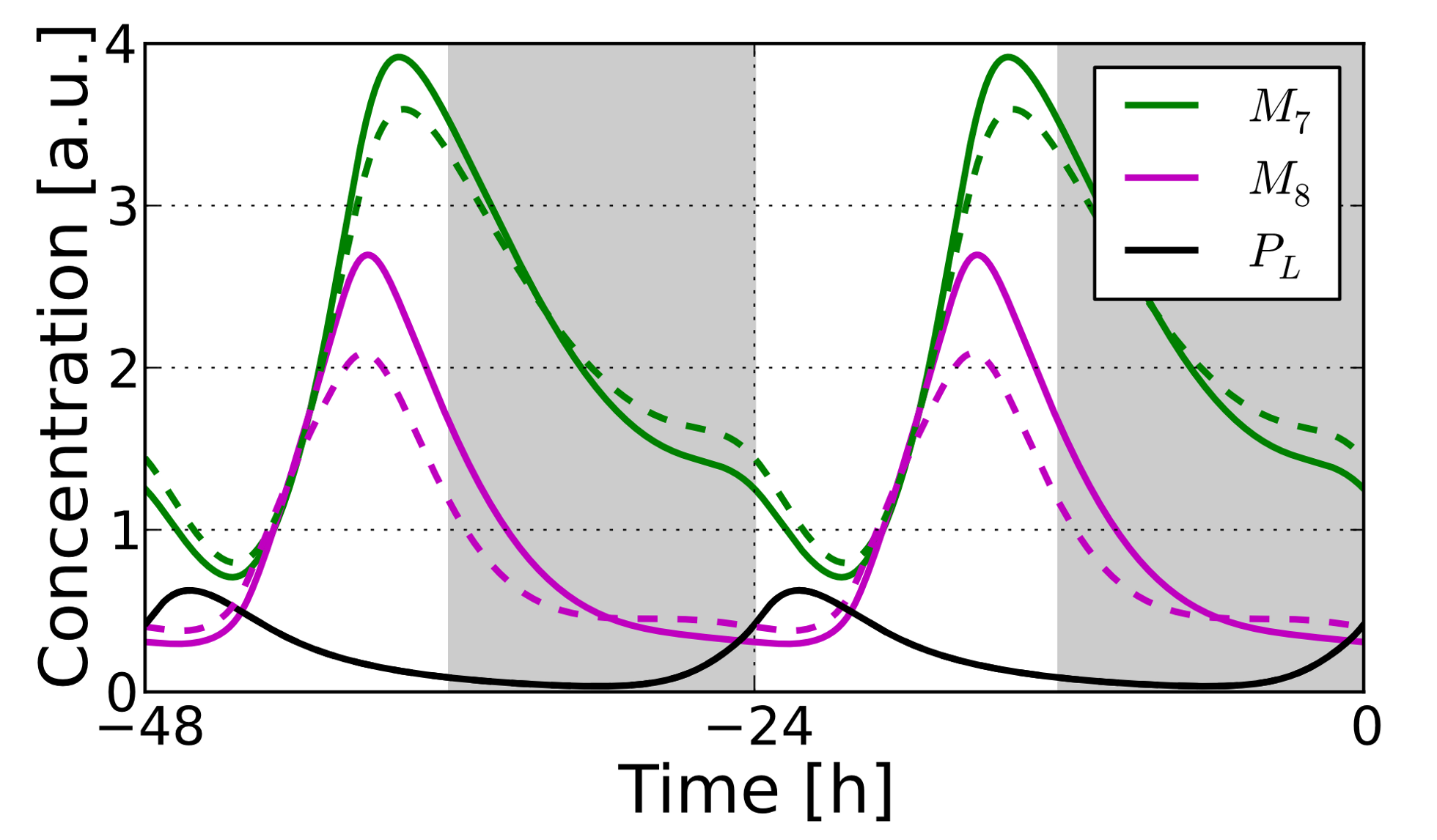

Supplement: Figure S4 — Dashed: Simulations for the optimal parameter set from Table 1, identical to those of Figure 2. Solid: Even if one adopts for AtGRP8 the same parameters as for AtGRP7 (see Table 1), apart from the constants connected to alternative splicing ( and ) and transcription kinetics (, , and ), the mRNA oscillations of AtGRP7 and AtGRP8 still behave qualitatively similar. In particular, the earlier peak of the AtGRP8 mRNA persists. (TIFF) [file pcbi.1002986.s004.tif]

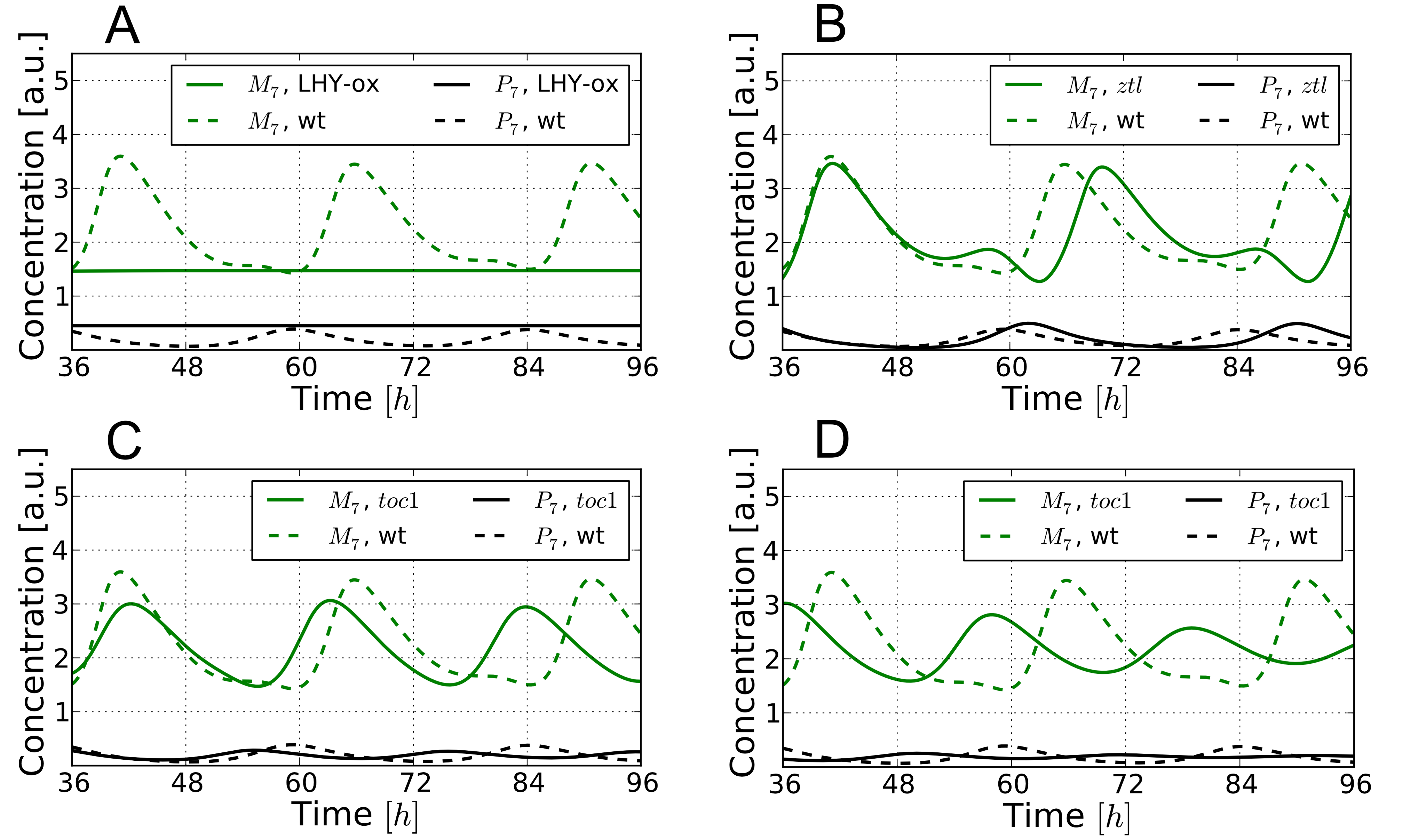

Supplement: Figure S5 — Simulations of the LHY overexpression (LHY-ox) mutant (A), ztl (B), and toc1 (D) null mutants as well as a hypothetical toc1 mutant (C), where the repression of PRR9 by TOC1 is neglected, as described in [11]. Dashed lines denote the wild type (wt) and continuous lines denote the mutant simulations of AtGRP7 mRNA (green) and LHY/CCA1 protein oscillations (black). (TIFF) [file pcbi.1002986.s005.tif]

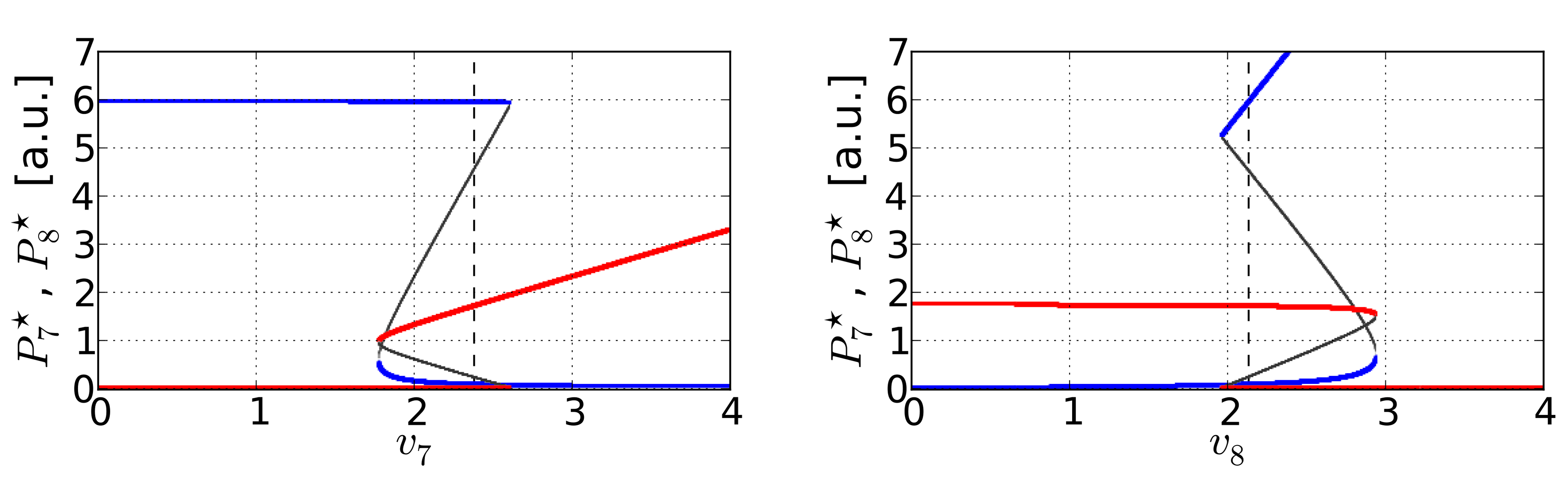

Supplement: Figure S6 — One parameter bifurcation diagrams of the maximal transcription rates (left) and (right), corresponding to the dashed lines in Figure 4 A/C/D. The protein concentration values and for stable fixed points are plotted in red and blue, respectively. Protein concentrations for unstable fixed points are kept in black. Dashed lines indicate the parameter values from the optimal parameter set of Table 1. (TIFF) [file pcbi.1002986.s006.tif]

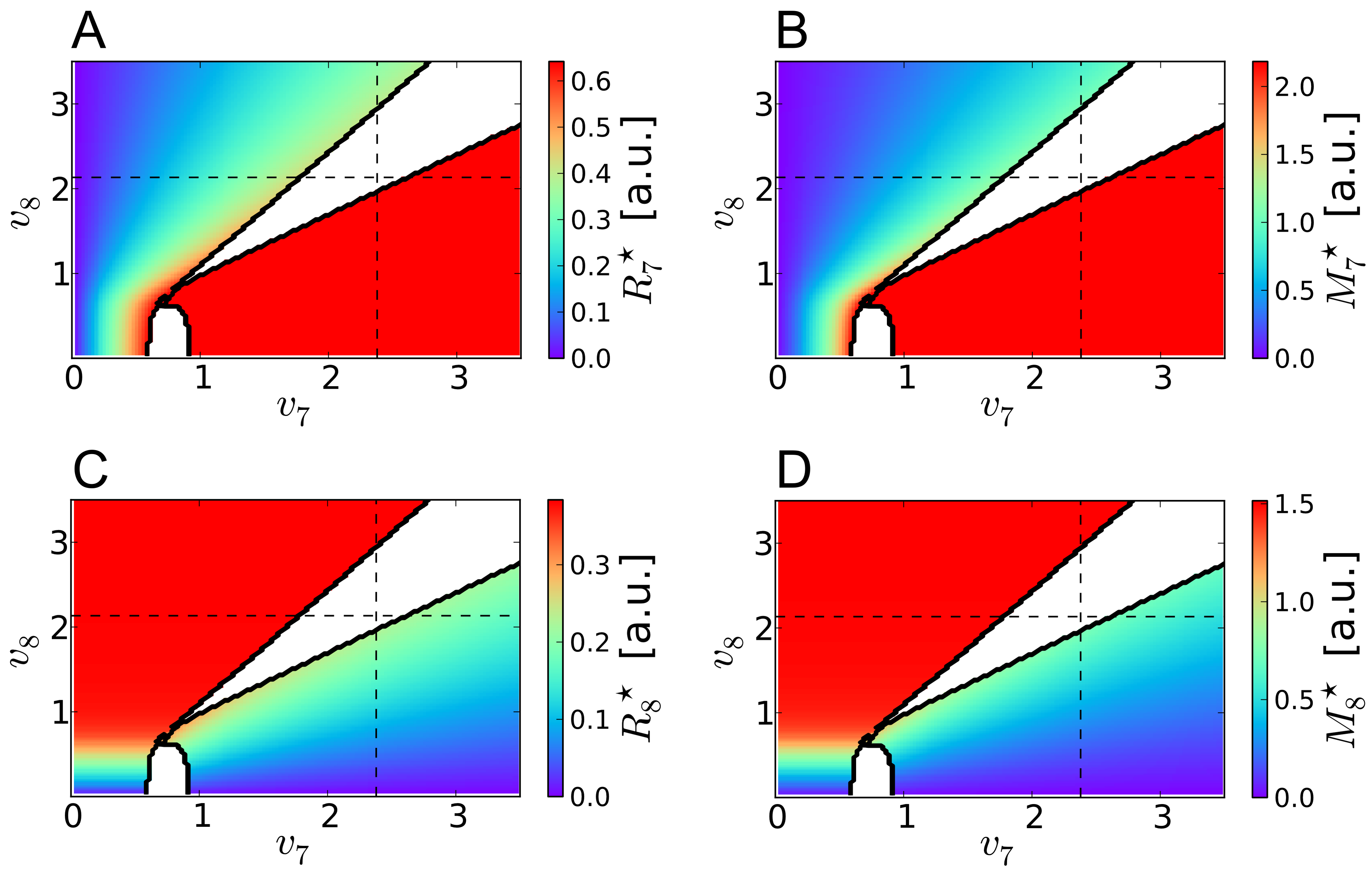

Supplement: Figure S7 — Analogously to Figures 4 C/D of the main text, we plotted the color-coded fixed point concentrations of the AtGRP7 pre-mRNA (A) and mRNA (B) as well as the AtGRP8 pre-mRNA (C) and mRNA (D) in the monostable areas of the – bifurcation diagram. The intersection of the dashed lines marks the optimal parameter set from Table 1. (TIFF) [file pcbi.1002986.s007.tif]

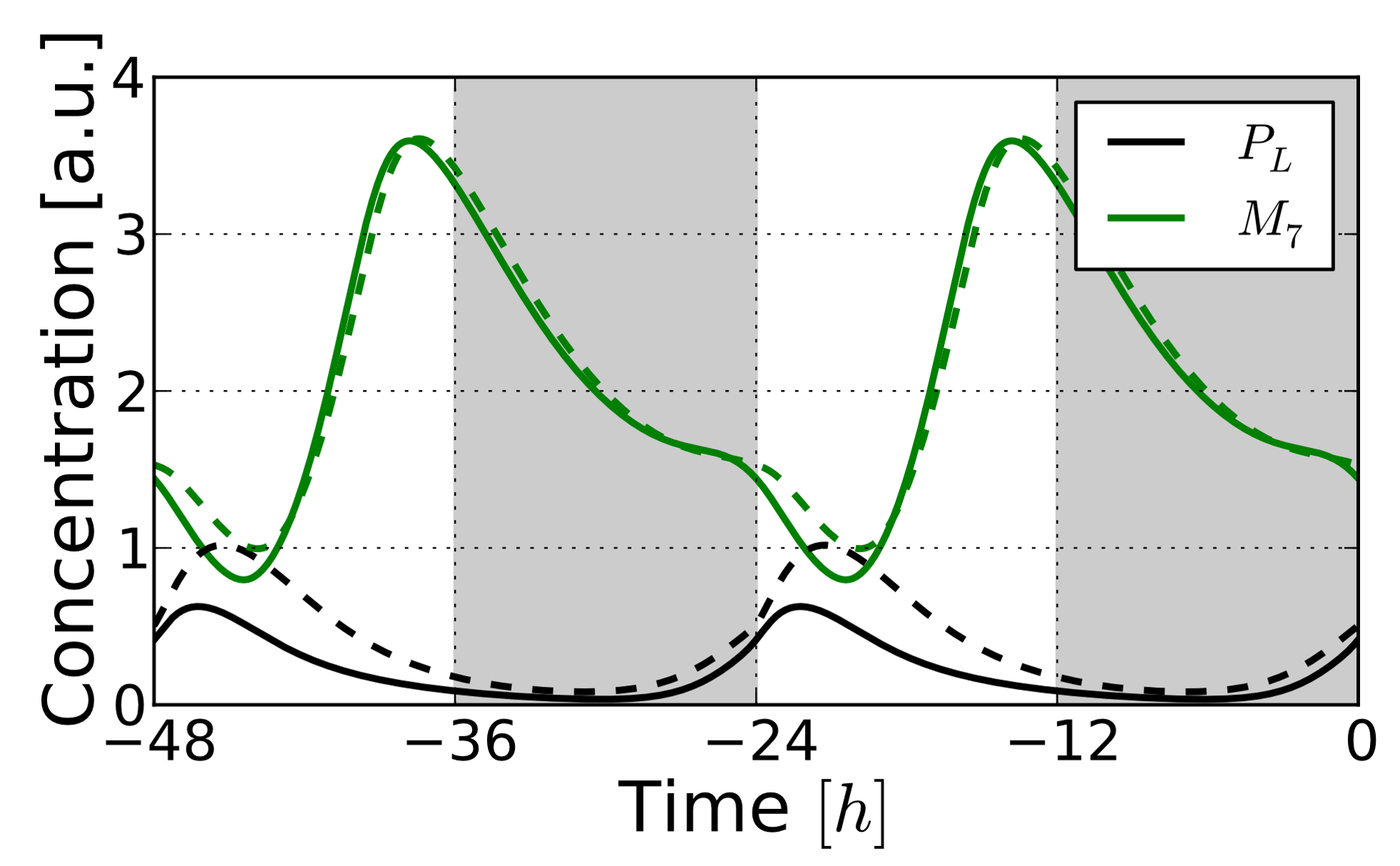

Supplement: Figure S8 — Solid: Reproduction of the results for and from Figure 2. Dashed: Corresponding results after replacing the original core oscillator model from [11] by the refined model from [13] and adapting the activation coefficients according to and . (TIFF) [file pcbi.1002986.s008.tif]

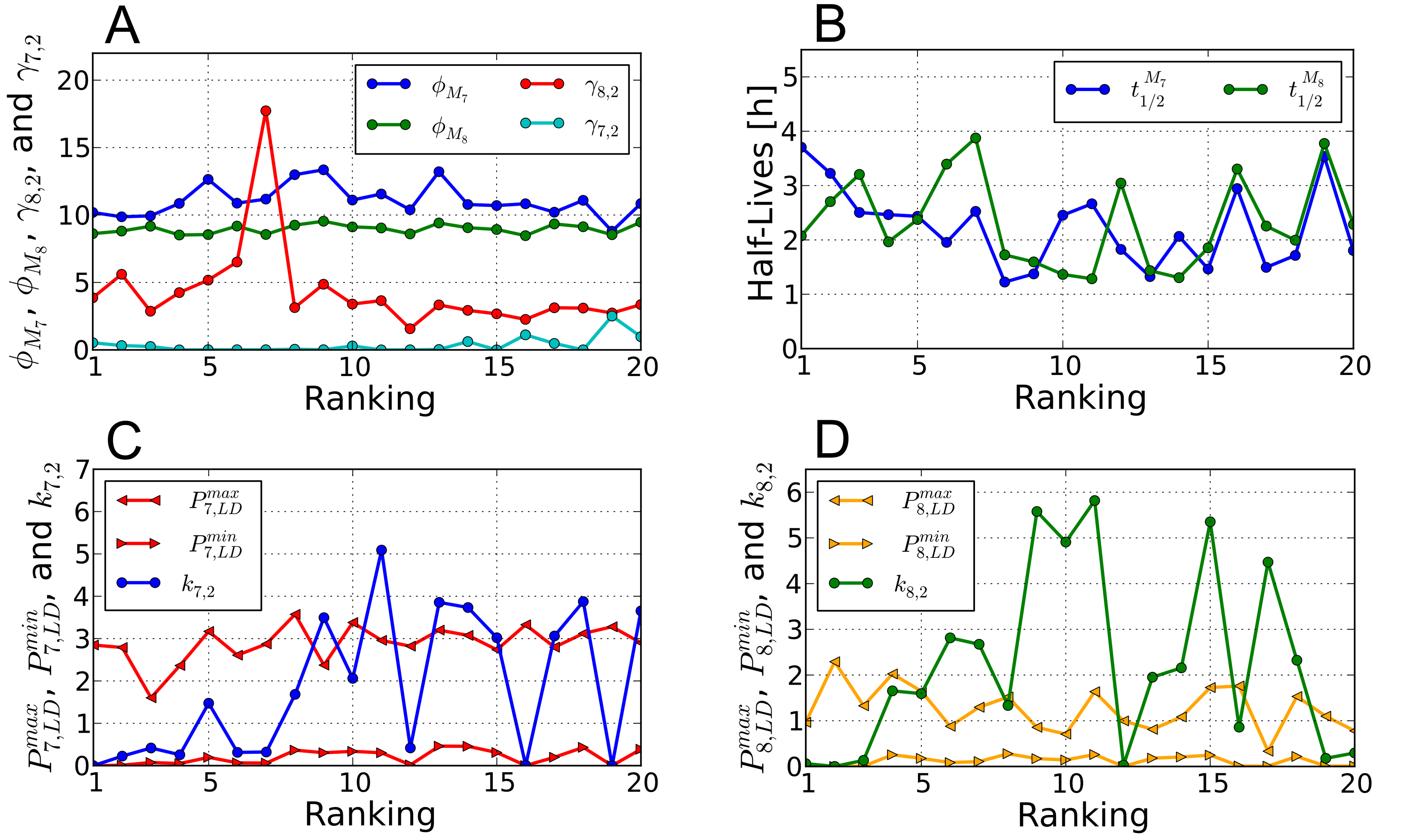

Supplement: Figure S9 — Four key features of the model dynamics (1)–(6) under 12h∶12h LD conditions for the optimal parameter set from Table 1 (Ranking) and for the 19 next best parameter sets (Ranking) resulting from the above described two-step optimization process with random initialization and subsequent evolutionary optimization. A) Two representative examples of the 20 (sub-)optimal parameter sets ( and ). As detailed in the main text, the observed general property indicates that the subordination of AtGRP8 to AtGRP7 is a robust feature of our optimization procedure. The experimentally observed earlier peak of AtGRP8 mRNA compared to AtGRP7 mRNA, i.e. (see section In silico waveforms and phases are consistent with the experimental data), is a further such robust feature. B) The half-lives and (see main text and Text S1 A) indicating that the shorter life-time of AtGRP8 mRNA compared to AtGRP7 mRNA is a less robust feature of our optimization procedure. Likewise, the depicted Michaelis constants and the peak () and trough values () of oscillations in C) () and D) () indicate that the saturation of AtGRP7 and AtGRP8 protein degradation is a less robust feature. (TIFF) [file pcbi.1002986.s009.tif]

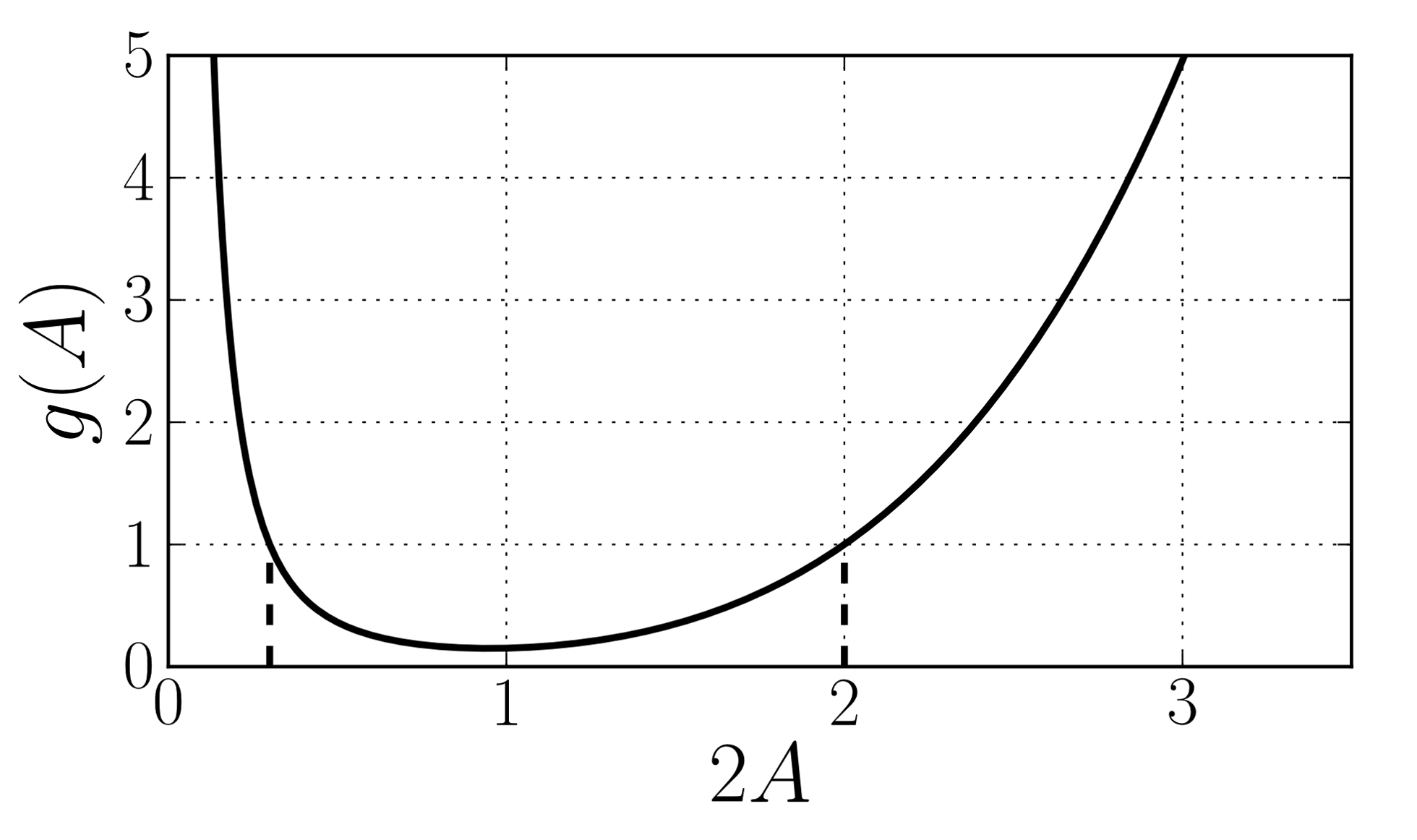

Supplement: Figure S10 — Function (see Text S1 A) is plotted versus different peak-trough-values . The peak-trough-values and , each leading to a cost function contribution of one, are indicated by vertical dashed lines. (TIFF) [file pcbi.1002986.s010.tif]
