# Supplementary material for: A Circadian Clock-Regulated Toggle Switch Explains AtGRP7 and AtGRP8 Oscillations in Arabidopsis thaliana
Source: PLoS Comput Biol. 2013 Mar 28;9(3):e1002986. doi: 10.1371/journal.pcbi.1002986 (PMC3610657; doi:10.1371/journal.pcbi.1002986)
Supplement: Text S1 — A) Detailed description of the cost function. B) Analysis of the one-component posttranscriptional feedback loop. C) Search for self-sustained oscillations. D) Search for noise-induced oscillations. (PDF) [file pcbi.1002986.s011.pdf]

Supplementary Text

A: Cost Function

Here, we provide the details of the cost function $f(\vec{p})$ which quantifies for every given parameter set $\vec{p}$ the deviations of the corresponding solution of equation (1)-(6) in the main text from the experimental findings 1-5 (see section *Modeling the AtGRP7-AtGRP8 Interlocked Feedback Loops* of the main text).

Similarly as in [1–6] we adopt an optimization technique based on a cost function of the form

$$f := f_{\mathrm{T_{LD}}} + f_{\mathrm{T_{LL}}} + f_{LC} + f_{\phi} + f_{A} + f_{\Delta} + f_{\nabla} + f_{t_{1/2}} \quad . \tag{1}$$

While all quantities in this equation are functions of $\vec{p}$, we henceforth omit those arguments $\vec{p}$ for the sake of simplicity. After solving the equations (1)-(6) for 27 days (14 days under 12h:12h LD and 13 days under LL conditions) as described in the main text, we neglected the first 236h in LD and the first 48h in LL as transients and applied the cost function to the remaining parts of the solutions. Accordingly, throughout this section $\langle\bullet\rangle_{\mathrm{LD}}$ and $\langle\bullet\rangle_{\mathrm{LL}}$ from now on denote the statistical average of a given feature $\bullet$ which repeatedly occurs within the considered time intervals $[236\mathrm{h}, 336\mathrm{h}]$ and $[384\mathrm{h}, 648\mathrm{h}]$, respectively. The standard deviations $\sigma[\bullet]_{\mathrm{LD}}$ and $\sigma[\bullet]_{\mathrm{LL}}$ as well as the coefficients of variation $\mathrm{CV}[\bullet]_{\mathrm{LD}} := \frac{\sigma[\bullet]_{\mathrm{LD}}}{\langle\bullet\rangle_{\mathrm{LD}}}$ and $\mathrm{CV}[\bullet]_{\mathrm{LL}} := \frac{\sigma[\bullet]_{\mathrm{LL}}}{\langle\bullet\rangle_{\mathrm{LL}}}$ of a given property $\bullet$ are defined analogously. In the following we will discuss each single term on the right hand side of equation (1) in detail.

$f_{\mathrm{T_{LD}}}$ & $f_{\mathrm{T_{LL}}}$ - Synchronized Oscillations 1: *AtGRP7* and *AtGRP8* mRNA adopt circadian oscillations in all diurnal data sets studied for wild type plants. We therefore require that they adopt the same period as the core oscillator both under LD and LL conditions.

The corresponding cost function term for the LD condition is defined as

$$f_{T_{\mathrm{LD}}} := \sum_{i\in\{7,8\}} \left(\frac{\Delta T_{\mathrm{LD},i}}{\delta T_{\mathrm{LD}}}\right)^2 \quad , \tag{2}$$

contributing a value smaller than unity if the deviation $\Delta T_{\mathrm{LD},i} := \langle T_{\mathrm{Core}}\rangle_{\mathrm{LD}} - \langle T_{\mathrm{Slave}}\rangle_{\mathrm{LD},i}$ of the slave oscillator's averaged period $\langle T_{\mathrm{Slave}}\rangle_{\mathrm{LD},i}$ from the averaged core oscillator's

period $\langle T_{\mathrm{Core}}\rangle_{\mathrm{LD}}$ is smaller than three minutes, i.e. $\delta T_{LD} := 0.05$h. The index $i = 7$ accounts for the period of *AtGRP7* mRNA and $i = 8$ for that of *AtGRP8* mRNA. The core oscillator period $\langle T_{\mathrm{Core}}\rangle_{\mathrm{LD}}$ is determined by the time difference between two successive maxima of the LHY/CCA1 protein oscillations $P_L(t)$. Likewise we define the periods $\langle T_{\mathrm{Slave}}\rangle_{\mathrm{LD},i}$ of *AtGRP7* and *AtGRP8* mRNA oscillations as the time difference between two consecutive maxima of the *AtGRP7* and *AtGRP8* mRNA concentrations $M_i(t)$, respectively. Solutions $M_i(t)$ showing monotonic behavior or only one maximum in the studied time span ($t \in [236\mathrm{h}, 336\mathrm{h}]$ or $t \in [384\mathrm{h}, 648\mathrm{h}]$) are sorted out and not further investigated, while e.g. chaotic or damped oscillations are still admitted.

Similarly, the cost function term for the LL condition is defined as

$$f_{T_{\mathrm{LL}}} := \sum_{i\in\{7,8\}} \left(\frac{\Delta T_{\mathrm{LL},i}}{\delta T_{\mathrm{LL}}}\right)^2 \tag{3}$$

with $\delta T_{LL} = 0.05$h.

f_{LC} **- Synchronized Oscillations 2:** Our circadian core oscillator model gives rise to periodic oscillations of $P_L(t)$ under 12h:12h LD as well as LL conditions [7]. We therefore require that the slave oscillator should also periodically oscillate under both circumstances. Similarly as in [1–3] we thus define

$$f_{LC} := \sum_{K\in\{M,P\}} \sum_{i\in\{7,8\}} \left[\frac{\mathrm{CV}\left[K_i^{\max}\right]_{\mathrm{LD}}}{0.05} + \frac{\mathrm{CV}\left[K_i^{\max}\right]_{\mathrm{LL}}}{0.05} + \frac{\mathrm{CV}\left[\phi_{K_i}\right]_{\mathrm{LD}}}{0.05}\right] \tag{4}$$

to quantify the entrainment of the slave oscillator by the core oscillator under LD and LL conditions. The first term gives a contribution smaller than unity if the relative variability around its mean value in LD of the concentration maxima $K_i^{\max}$ of a given oscillating variable K_i is smaller than 5%. Likewise, the second term accounts for the LL conditions and the third term for the variability of the phases ϕ_{K_i} (see main text, section "Comparison with Experimental Results and Predictions") among successive LD-cycles.

In summary the term (4) thus penalizes significant variations in phase or height of the oscillation maxima (e.g. chaotic solutions or damped oscillations) while the terms (2) and (3) impose the periodicity of the core oscillator to the slave.

f_ϕ **- Experimentally observed Phases:** The model of our core oscillator was optimized for 12h:12h LD experimental entrainment conditions [7]. We therefore used the data sets from the DIURNAL data bank experimentally obtained under those 12h:12h LD conditions as well. The phase of the *AtGRP7* mRNA oscillations was estimated as $\phi_{M_7}^{\mathrm{exp}} = \mathrm{zt}\,10\mathrm{h}$ [8]. The *AtGRP8* mRNA oscillations precede those of *AtGRP7* by two hours [8], similar to what was found in studies for other entrainment conditions [8, 9], so we set $\phi_{M_8}^{\mathrm{exp}} = \mathrm{zt}\,8\mathrm{h}$. There is, to the best of our knowledge, no published data on *At*GRP7 or *At*GRP8 protein concentrations for 12h:12h LD entrainment conditions. In [10] one can find data on protein concentrations under 8h:16h LD entrainment conditions which revealed that *At*GRP7 protein peaks approximately four hours after its mRNA. We used this as a gross approximation also under 12h:12h LD conditions and set $\phi_{P_7}^{\mathrm{exp}} = \phi_{M_7}^{\mathrm{exp}} + 4\mathrm{h}$ and $\phi_{P_8}^{\mathrm{exp}} = \phi_{M_8}^{\mathrm{exp}} + 4\mathrm{h}$, respectively.

f_ϕ then contains four summands and reads as

$$f_\phi := \sum_{K\in\{M,P\}} \sum_{i\in\{7,8\}} \left(\frac{\langle \phi_{K_i} \rangle_{\mathrm{LD}} - \phi_{K_i}^{\mathrm{exp}}}{\delta\phi_{K_i}} \right)^2 \quad , \tag{5}$$

where the different reliability of the data is reflected by our error tolerances $\delta\phi_{M_7} = \delta\phi_{M_8} = 0.5\mathrm{h}$ and $\delta\phi_{P_7} = \delta\phi_{P_8} = 1.0\mathrm{h}$, respectively.

f_A **- Amplitude:** In view of [8] we expect that the mRNAs and proteins of *At*GRP7 and *At*GRP8 exhibit concentrations (number of molecules per volume within the pertinent cell compartment) which are very roughly speaking of the same order of magnitude as the concentrations $M_L(t)$ and $P_L(t)$ of LHY/CCA1. Therefore we defined the summand f_A related to the amplitude A of the oscillations as

$$f_A := \sum_{K\in\{M,P\}} \sum_{i\in\{7,8\}} g(A), \tag{6}$$

where $g(A)$ is depicted in Figure S10. This function $g(A)$ has been obtained according to the following considerations: Starting with the Ansatz

$$g(A) := \left(\frac{c_{\mathrm{min}}}{2A_{K_i}} \right)^2 + \left(2A_{K_i} c_{\mathrm{max}}\right)^4 , \tag{7}$$

the parameters $c_{\min}$ and $c_{\max}$ were chosen so as to generate $g(A)$ values smaller than one for all peak-trough-values $2A$ within the heuristically predetermined range between 0.3 and 2.0, resulting in $c_{\min} \approx 0.30$, $c_{\max} \approx 2.01$.

f_Δ & f_∇ - Waveform: Next we turn to the waveform of the experimentally observed *AtGRP7* and *AtGRP8* mRNA as well as protein oscillations. Experimental time traces of *AtGRP7* and *AtGRP8* mRNA show non-sinusoidal behavior [8, 10]. After being at its trough value, *AtGRP7* and *AtGRP8* mRNA quickly recovers, typically within four hours, to its maximal value, and needs a slightly longer time to reach its trough value again, creating nevertheless sharp mRNA peaks. Experimentally observed *At*GRP7 protein time traces [10], anti-phasic to its mRNA, are much broader and stay at their trough value much shorter. Similarly as in [1–3] we thus define

$$
\begin{aligned}
f_\Delta := & \sum_{i\in\{7,8\}} \sum_{\tau_M\in\{-4\mathrm{h},6\mathrm{h}\}} \left(\frac{2/3\langle M_i^{\max}\rangle\rangle_{\mathrm{LD}}}{\langle M_i^{\max} - M_i^{\max+\tau_M}\rangle_{\mathrm{LD}}} \right)^2 \\
& + \sum_{i\in\{7,8\}} \sum_{\tau_M\in\{-4\mathrm{h},6\mathrm{h}\}} \left(\frac{2/3\langle M_i^{\max}\rangle_{\mathrm{LL}}}{\langle M_i^{\max} - M_i^{\max+\tau_M}\rangle_{\mathrm{LL}}} \right)^2 \\
& + \sum_{\tau_P\in\{-12\mathrm{h},8\mathrm{h}\}} \left(\frac{2/3\langle P_7^{\max}\rangle_{\mathrm{LL}}}{\langle P_7^{\max} - P_7^{\max+\tau_P}\rangle_{\mathrm{LL}}} \right)^2 \quad ,
\end{aligned}
\tag{8}
$$

where $M_i^{\max+\tau_M}$ denotes the M_i concentration τ_M hours before or after its concentration maximum and likewise for $P_7^{\max+\tau_P}$. Generally speaking f_Δ thus penalizes solutions that do not show the experimentally observed sharp mRNA peak and broad protein peak, respectively. More precisely the first term on the right hand side of equation (8) contributes a value smaller than unity for solutions of the $M_7(t)$ and $M_8(t)$ oscillations in LD conditions that drop at least by 2/3 of their maximum value within 4 hours before and 6 hours after the respective peak time, and larger than unity otherwise. The second term works in the same manner for LL conditions. Each summand of the third term similarly contributes a value smaller than unity for solutions of the $P_7(t)$ oscillations in LL conditions, that drop at least by 2/3 of their maximum value within 12 hours before and 8 hours after the respective peak time, consistent with the broad protein oscillations observed in [10].

The term f_∇ in (1) accounts for the trough-broadness of the *AtGRP7* and *AtGRP8*

mRNA oscillations in LL conditions and is defined as

$$f_\nabla := \sum_{i\in\{7,8\}} \left(\frac{\nabla_i - \nabla_i^{\exp}}{\delta\nabla}\right)^2 \quad . \tag{9}$$

Here ∇_7 is defined as the time the *AtGRP7* mRNA concentration exceeds its minimal value by less than 10 per cent of the peak-trough-difference and similarly for ∇_8. The corresponding experimental quantities $\nabla_i^{\exp}$ are quite noisy, ranging from 8 [8] to 12 [10] hours. We therefore set $\nabla_i^{\exp} = 10\text{h}$ and $\delta\nabla = 2\text{h}$.

$f_{t_{1/2}}$ **- Half-Life:** In experiments measuring the *AtGRP7* mRNA half-life [11,12], plants were grown under 16h:8h LD long-day entrainment conditions and were transferred to a medium supplemented with $150\,\mu\text{g}\,\text{ml}^{-1}$ cordycepin, two hours before expecting the mRNA peak, in order to suppress transcription. Afterwards, the mRNA abundance was measured with one-hour time intervals, observing that the mRNA is reduced to 50% of its original amount within 3-4 hours. We therefore set $t_{1/2}^{\exp} = 3.5\text{h}$ with an error tolerance of $\delta t_{1/2} = 0.5\text{h}$. We followed the experimental protocol in our simulations exactly. First, we inhibited the transcription of *AtGRP7* and *AtGRP8* in the model by setting v_7 and v_8 in equations (1) and (4) of the main text to zero. Since the production of *AtGRP7* mRNA depends on the normal splicing of its pre-mRNA and since its degradation kinetics are of Michaelis-Menten type, its half-life will depend on the systems initial conditions. This initial concentrations of the slave oscillator were set, in analogy to experiments, to the values two hours before the maximum of the *AtGRP7* mRNA oscillation is expected within the last entrainment cycle, i.e. for $t \in [312\text{h}, 336\text{h}]$. Given these initial conditions, we solved the resulting equations for a maximal time of $\tau_{\max} = 16\text{h}$ and define the half-life $t_{1/2}$ as the time a given concentration needs to decline to the half of its initial value (see Figure S3 A/B). The summand $f_{t_{1/2}}$ then reads as

$$f_{t_{1/2}} = \left(\frac{t_{1/2} - t_{1/2}^{\exp}}{\delta t_{1/2}}\right)^2 \quad . \tag{10}$$

In case the system did not decay to half of the initial *AtGRP7* mRNA concentration within $\tau_{\max} = 16\text{h}$, we set $t_{1/2} = \tau_{\max}$, therefore defining a maximal contribution to the cost function $f_{t_{1/2}}^{\max} = \left(\frac{16\text{h}-3.5\text{h}}{0.5\text{h}}\right)^2 = 625$.

As there is no experimental data available on the half-life of *AtGRP8* mRNA, the cost-function lacks a corresponding term. In [12] the half-life of the alternatively spliced mRNA of *AtGRP7* was estimated as 0.5h. Since this alternatively spliced mRNA was not assumed to be able of producing functional protein, it will therefore not feed back to the system described by equations (1)-(6) in the main text and a corresponding cost function-term can therefore be neglected.

B: The One-Component Posttranscriptional Feedback Loop

According to equations (1)-(6) in the main text, the single posttranscriptional *At*GRP7 feedback loop model without interlocking to *At*GRP8 (which is equivalent to setting the parameter $\gamma_{7,2}$ to zero) reads as

$$\dot{R}_7(t) = \frac{v_7}{1 - \left(\frac{P_L(t)}{h_7}\right)^{i_7}} - (\gamma_{7,1} P_7(t) + \delta_7) R_7(t) \tag{11}$$

$$\dot{M}_7(t) = \delta_7 R_7(t) - \frac{m_{7,1} M_7(t)}{k_{7,1} + M_7(t)} \tag{12}$$

$$\dot{P}_7(t) = \xi_7 M_7(t) - \frac{m_{7,2} P_7(t)}{k_{7,2} + P_7(t)} \quad . \tag{13}$$

In the undriven case ($P_L(t) = 0$ for all t), the steady state concentrations $\vec{x}^\star = (R_7^\star, M_7^\star, P_7^\star)^T$ fulfilling the fixed point conditions $\dot{\vec{x}}|_{\vec{x}^\star} = 0$ are

$$P_7^{\star,\pm} = \pm \sqrt{\frac{v_7 \delta_7 \xi_7 k_{7,1} k_{7,2}}{\gamma_{7,1} m_{7,1} m_{7,2}} + \alpha^2} - \alpha \tag{14}$$

$$M_7^{\star,\pm} = \frac{m_{7,2} P_7^{\star,\pm}}{\xi_7 (k_{7,2} + P_7^{\star,\pm})} \tag{15}$$

$$R_7^{\star,\pm} = \frac{m_{7,1} M_7^{\star,\pm}}{\delta_7 (k_{7,1} + M_7^{\star,\pm})} \quad , \tag{16}$$

where we introduced the abbreviation

$$\alpha = \frac{1}{2} \frac{\delta_7 m_{7,1} m_{7,2} - v_7 \delta_7 \xi_7 k_{7,1} - v_7 \delta_7 m_{7,2}}{\gamma_{7,1} m_{7,1} m_{7,2}} \quad . \tag{17}$$

$P_7^{\star,+}$ is always positive and $P_7^{\star,-}$ is always negative if we assume that all kinetic parameters are positive. $R_7^{\star,+}$ and $M_7^{\star,+}$ are then also positive and the sign of $R_7^{\star,-}$ and $M_7^{\star,-}$ can be either positive or negative depending on the values of $k_{7,1}$ and $k_{7,2}$, respectively. However this analysis shows that the system is not able to show bistability in the positive regime for any parameterization where the kinetic constants are assumed to be positive.

C: Search for Self-Sustained Oscillations

We tried to find self-sustained autonomous oscillation in our decoupled slave oscillator system, i.e. $P_L(t) = 0$ in equations (1)-(6) of the main text, by modifying the kinetic parameters of Table 1 without changing our model itself. To this end we searched for autonomous oscillations in the neighborhood of the parameter set in Table 1 by means of a gradient descent method that drives the real part of the complex conjugated pair of eigenvalues of the dynamical fixed point across the imaginary axis, corresponding to a Hopf bifurcation of the fixed point into a periodic solution. Remarkably enough, this algorithm indeed generated self-sustaining oscillations (see Figure 7 B of the main text) by only changing the protein degradation rates $m_{7,2}$ and $m_{8,2}$, while leaving all other kinetic parameters unchanged. The period of these oscillations is mainly determined by the protein degradation rate $m_{7,2}$ of *At*GRP7, exhibiting a decreasing period with increasing $m_{7,2}$, but still remaining below the experimentally observed circadian periodicity. In doing so, we restricted our search to a sufficiently small neighborhood of the reference parameter set from Table 1 in order to maintain a reasonably good agreement with the experimental facts 1-5 from section *Parameter Estimation* of the main text. However, even within this neighborhood all parameter sets exhibiting oscillations actually disagreed already quite notably from the experimental facts 1-5 (see section *Parameter Estimation* of the main text).

D: Search for Noise-Induced Oscillations

Following [13,14] we model concentration fluctuations by adding noise in equations (1)-(6) of the main text, i.e. equation (7) of the main text now takes the form

$$\dot{x}_k(t) = g_k\left(t, \vec{x}(t), \vec{p}\right) + \sigma x_k(t)\zeta_k(t), \qquad (18)$$

where σ quantifies the noise strengths and $\zeta_k(t)$ are independent, delta-correlated Gaussian white noises of zero mean. The noisy dynamics (18) were tackled by using *XPPAUT* (Version 6.11). Figure 7 C demonstrates the possibility of noise-induced self-sustained oscillations for suitable noise strengths σ. For the specific σ value from Figure 7 C a spectral analysis reveals a main peak around $T \approx 21.4\,\mathrm{h}$, a period close to the experimentally observed circadian rhythmicity.

References

1. Locke J, Millar A, Turner M (2005) Modelling genetic networks with noisy and varied experimental data: the circadian clock in *Arabidopsis thaliana*. Journal of Theoretical Biology 234: 383–393.

2. Locke JCW, Southern MM, Kozma-Bognar L, Hibberd V, Brown PE, et al. (2005) Extension of a genetic network model by iterative experimentation and mathematical analysis. Mol Syst Biol 1:2005.0013.

3. Locke JCW, Kozma-Bognar L, Gould PD, Feher B, Kevei E, et al. (2006) Experimental validation of a predicted feedback loop in the multi-oscillator clock of *Arabidopsis thaliana*. Mol Syst Biol 2:59.

4. Zeilinger MN, Farre EM, Taylor SR, Kay SA, Doyle FJ (2006) A novel computational model of the circadian clock in arabidopsis that incorporates PRR7 and PRR9. Mol Syst Biol 2:58.

5. Locke JC, Westermark PO, Kramer A, Herzel H (2008) Global parameter search reveals design principles of the mammalian circadian clock. BMC Systems Biology 2: 22.

6. Akman OE, Locke JCW, Tang S, Carré I, Millar AJ, et al. (2008) Isoform switching facilitates period control in the neurospora crassa circadian clock. Molecular Systems Biology 4.

7. Pokhilko A, Hodge SK, Stratford K, Knox K, Edwards KD, et al. (2010) Data assimilation constrains new connections and components in a complex, eukaryotic circadian clock model. Mol Syst Biol 6:416.

8. Mockler TC, Michael TP, Priest HD, Shen R, Sullivan CM, et al. (2007) The diurnal project: Diurnal and circadian expression profiling, model-based pattern matching, and promoter analysis. Cold Spring Harbor Symposia on Quantitative Biology 72: 353–363.

9. Schöning JC, Streitner C, Meyer IM, Gao Y, Staiger D (2008) Reciprocal regulation of glycine-rich RNA-binding proteins via an interlocked feedback loop coupling alternative splicing to nonsense-mediated decay in arabidopsis. Nucl Acids Res 36: 6977–6987.

10. Heintzen C, Nater M, Apel K, Staiger D (1997) AtGRP7, a nuclear RNA-binding protein as a component of a circadian-regulated negative feedback loop in *Arabidopsis thaliana*. Proc Natl Acad Sci U S A 94: 8515–8520.

11. Holtorf H, Schöb H, Kunz C, Waldvogel R, Meins F (1999) Stochastic and nonstochastic post-transcriptional silencing of chitinase and β-1,3-glucanase genes involves increased RNA turnoverpossible role for ribosome-independent RNA degradation. The Plant Cell 11: 471–483.

12. Staiger D, Zecca L, Kirk DAW, Apel K, Eckstein L (2003) The circadian clock regulated RNA-binding protein AtGRP7 autoregulates its expression by influencing alternative splicing of its own pre-mRNA. The Plant Journal 33: 361–371.

13. Mackey MC, Tyran-Kamiska M, Yvinec R (2011) Molecular distributions in gene regulatory dynamics. Journal of Theoretical Biology 274: 84–96.

14. Gehrmann E, Gläßer C, Jin Y, Sendhoff B, Drossel B, et al. (2011) Robustness of glycolysis in yeast to internal and external noise. Physical Review E 84: 021913.
